# Supplementary material for: Genetic diversity and phylogeography of Phlebotomus argentipes (Diptera: Psychodidae, Phlebotominae), using COI and ND4 mitochondrial gene sequences
Source: PLoS One. 2023 Dec 29;18(12):e0296286. doi: 10.1371/journal.pone.0296286 (PMC10756540; doi:10.1371/journal.pone.0296286)

Supplementary Table 2- Identified haplogroups in *COI* study data set

| Haplogroup ID | Network of the haplogroup | Haplotypes in the haplogroup |
| --- | --- | --- |
| **I** | **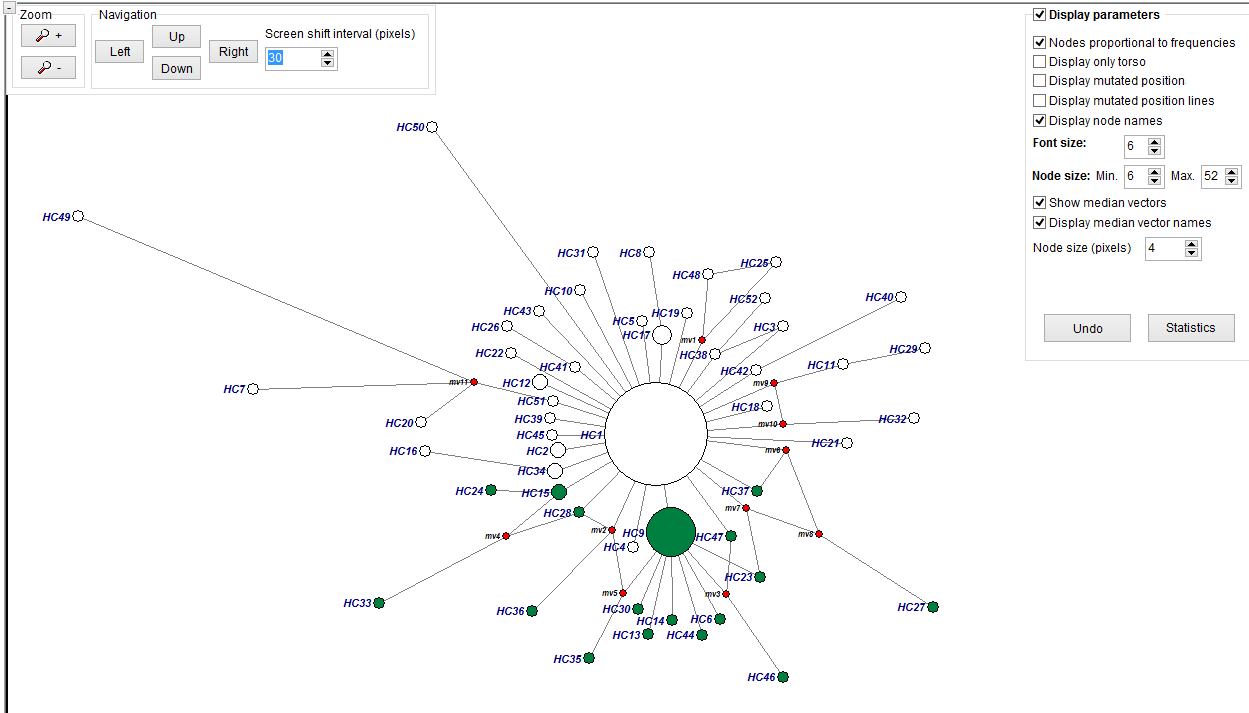** | ***HC6, HC13, HC14, HC15, HC24, HC23, HC27, HC28, HC30, HC33, HC35, HC37, HC36, HC44, HC46, HC47*** |
| **II** | **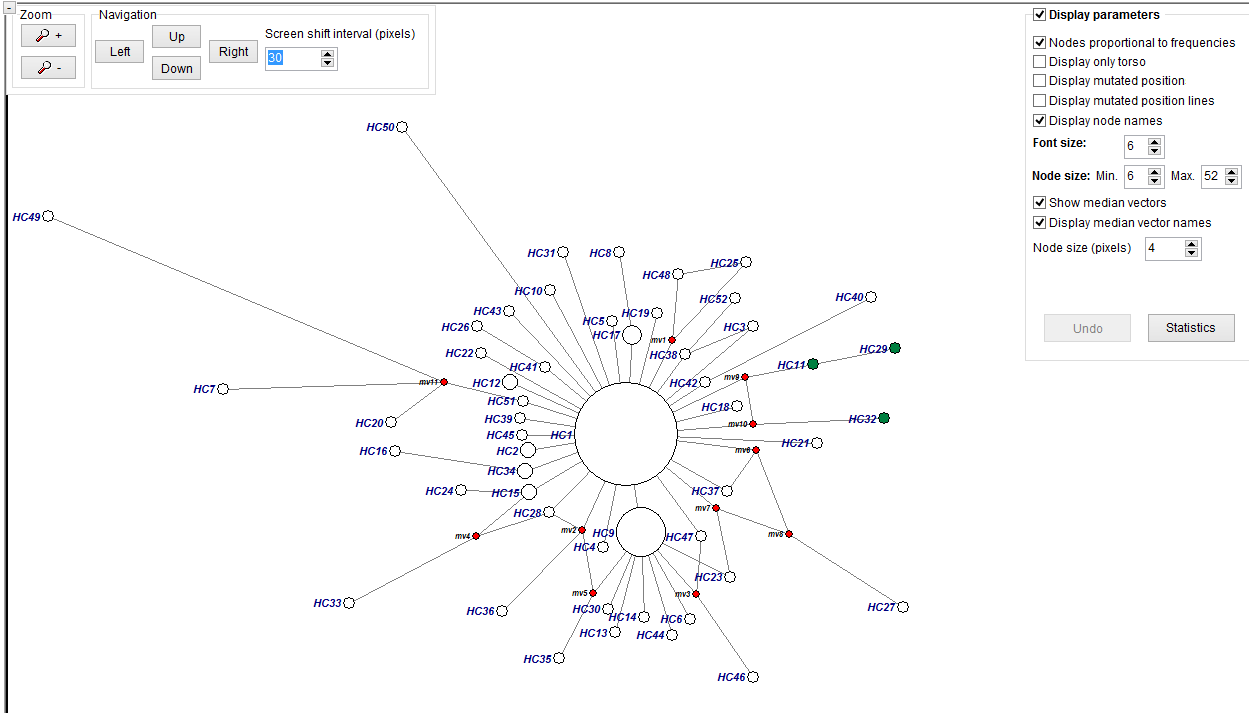** | ***HC11, HC29, HC32*** |
| **III** | **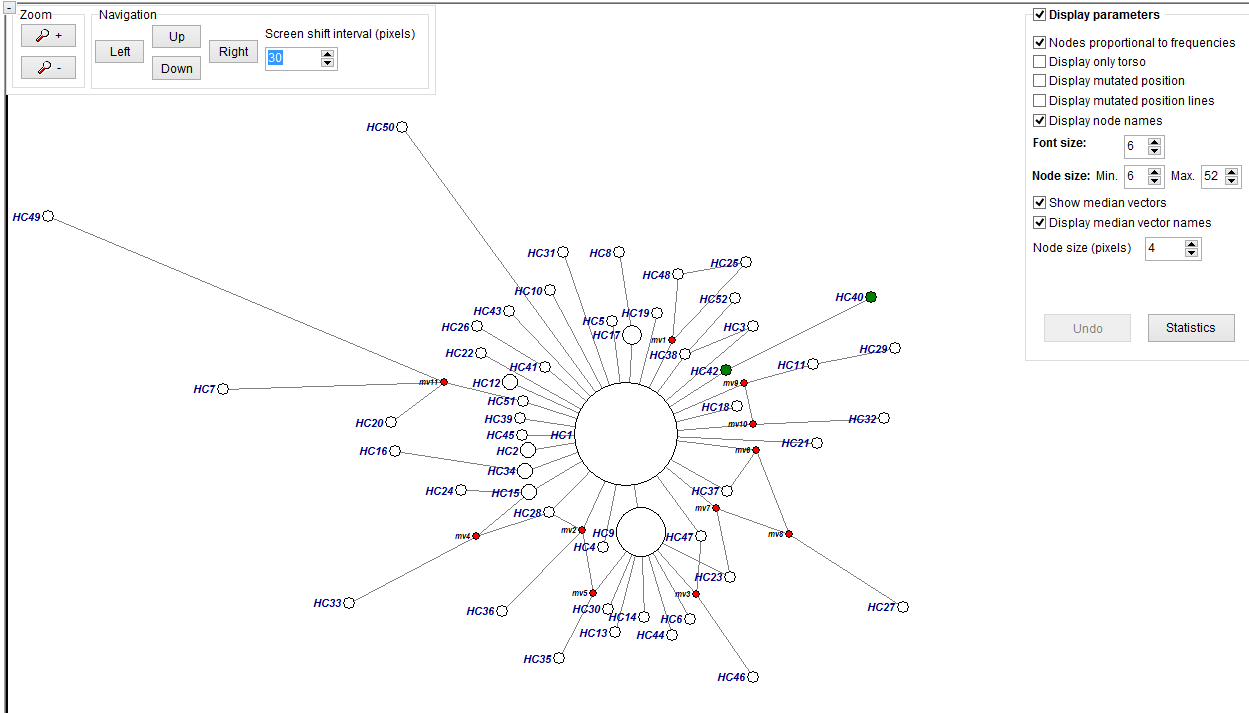** | ***HC40, HC42*** |
| **IV** | **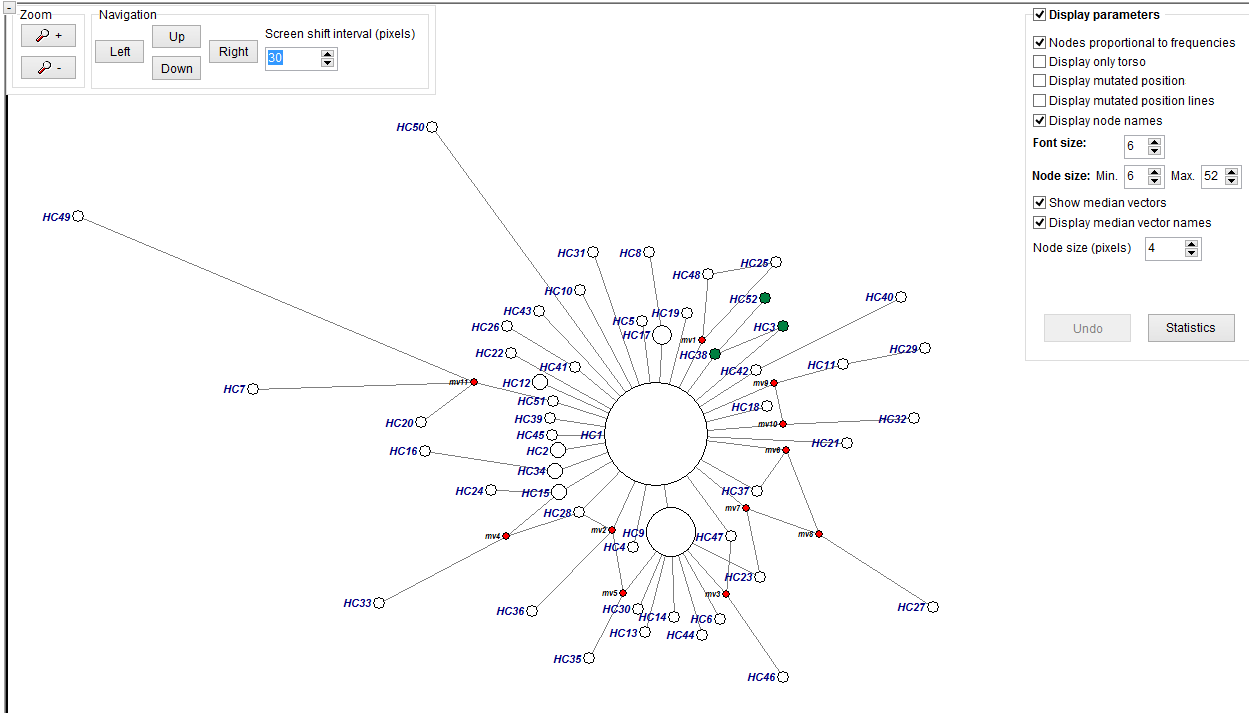** | ***HC3, HC38, HC52*** |
| **V** | **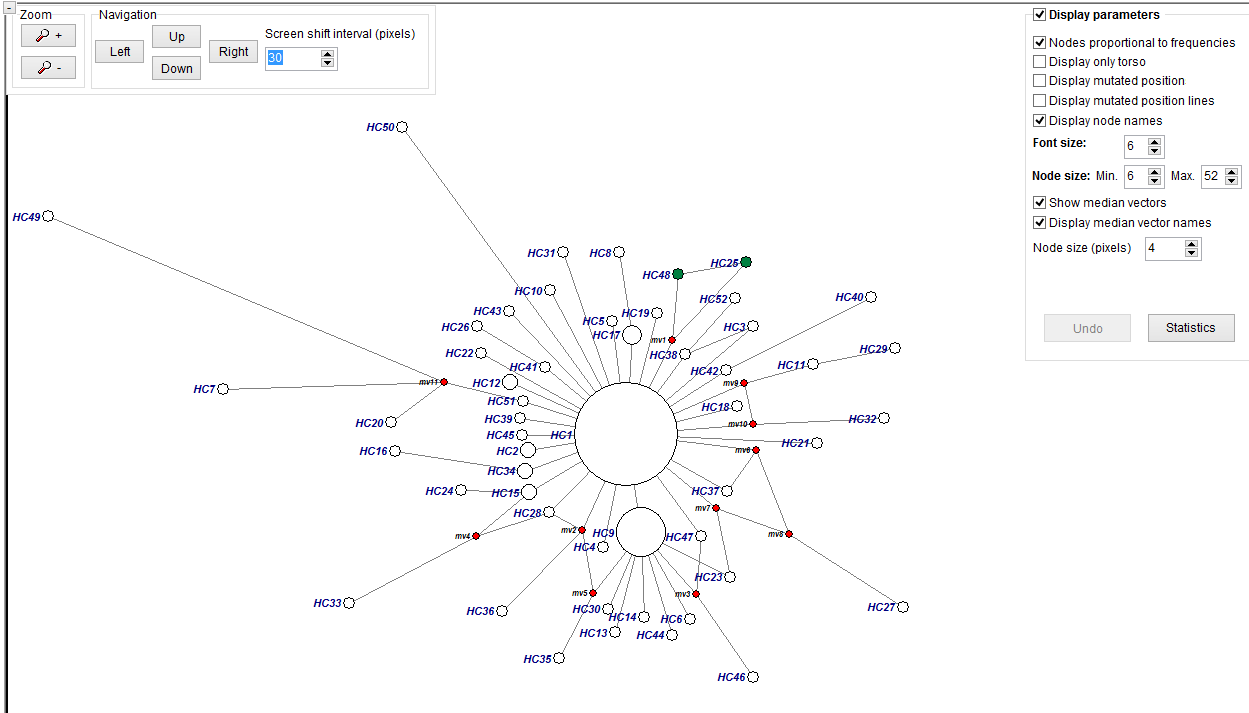** | ***HC25, HC48*** |
| **VI** | 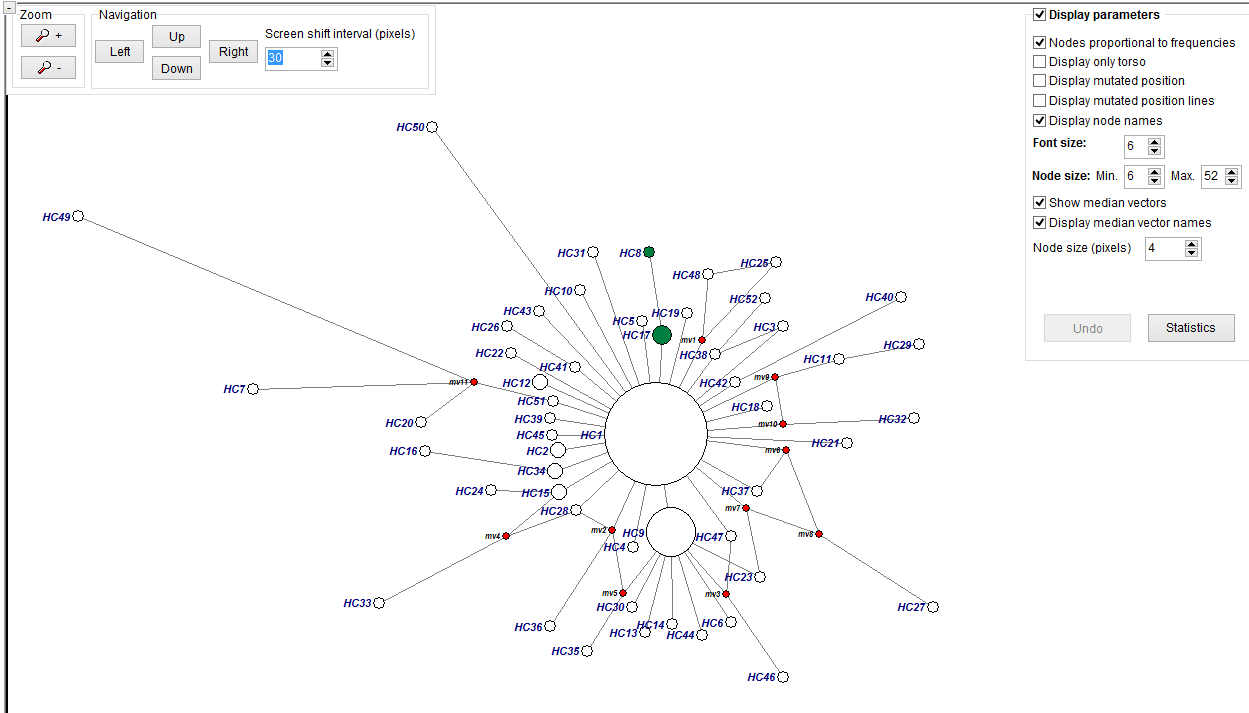 | ***HC8, HC19*** |
| **VII** | **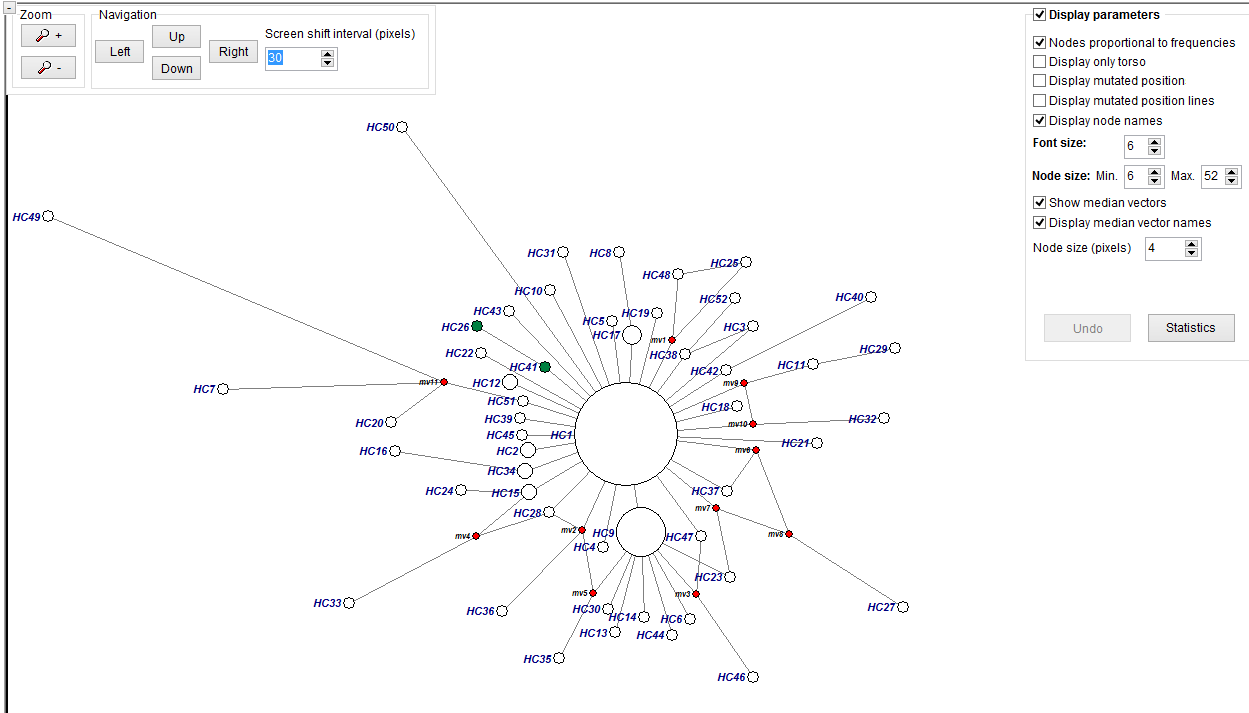** | ***HC26, HC41*** |
| **VIII** | **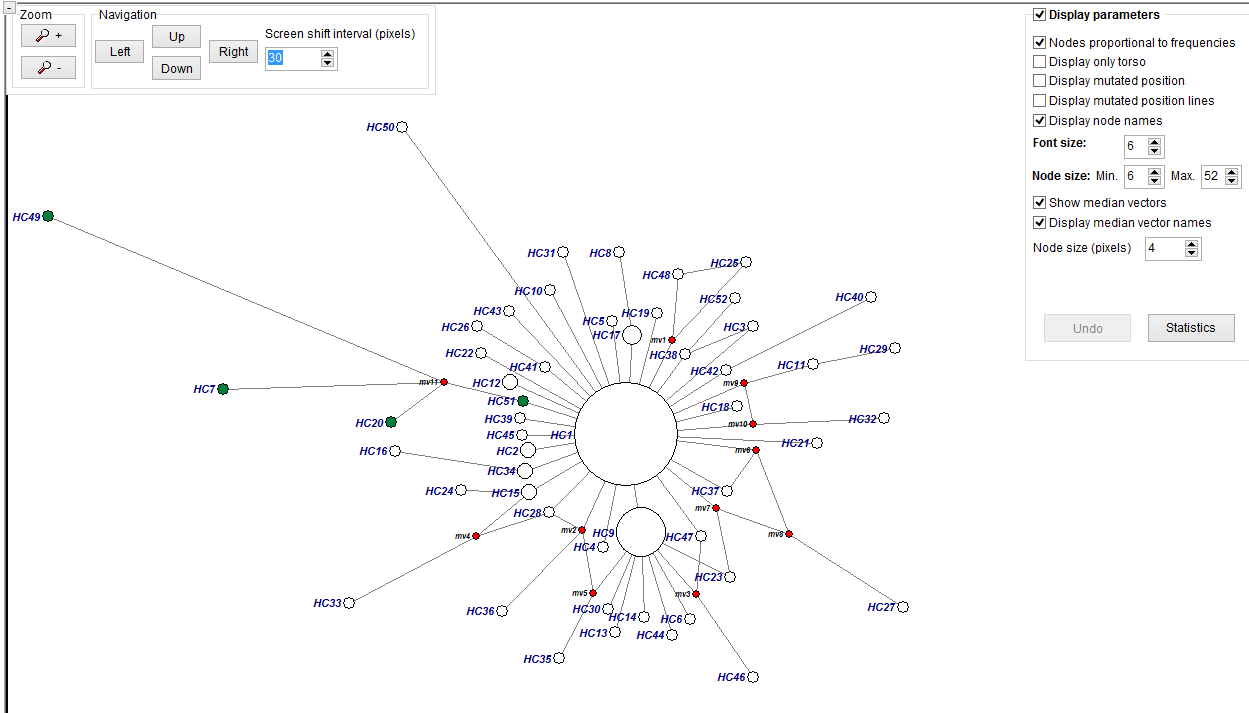** | ***HC7, HC20, HC49, HC51,*** |
| **IX** | **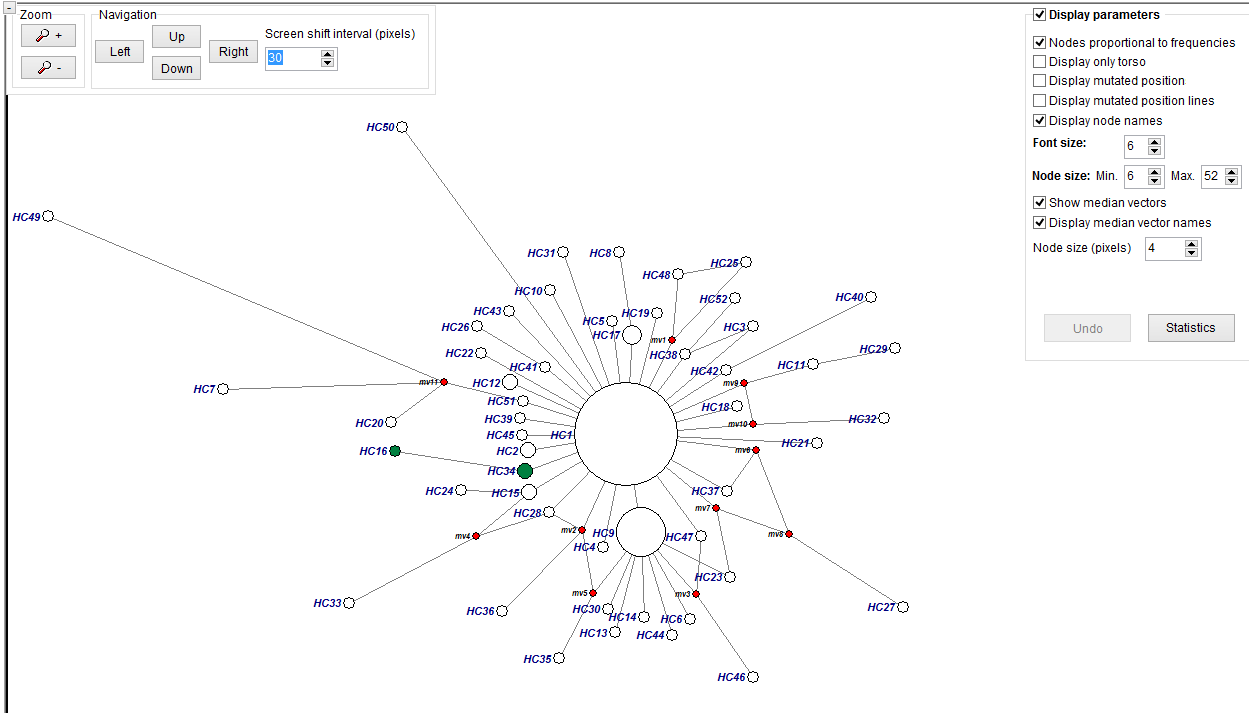** | ***HC16, HC34*** |


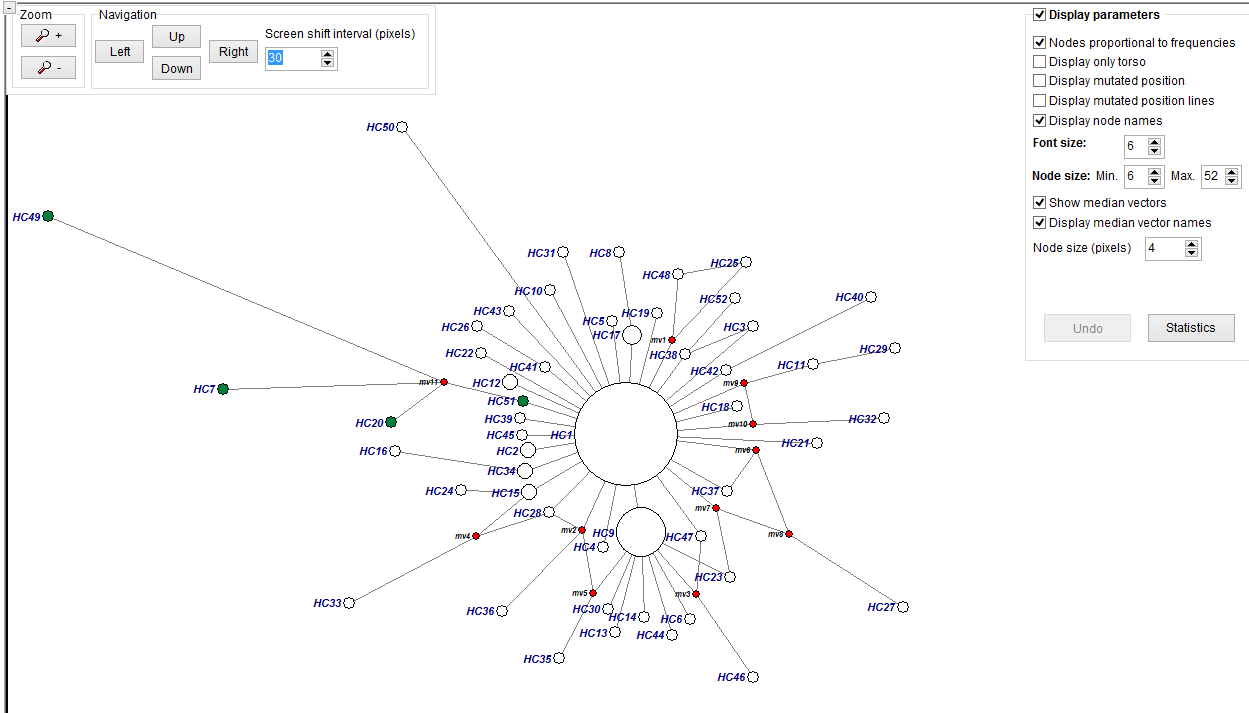

Supplement: S2 Table — Haplogroups identified from the COI study alignment of P. argentipes, along with corresponding haplotypes within each group. (DOC) [file pone.0296286.s002.doc]
