# Supplementary material for: Genetic diversity and phylogeography of Phlebotomus argentipes (Diptera: Psychodidae, Phlebotominae), using COI and ND4 mitochondrial gene sequences
Source: PLoS One. 2023 Dec 29;18(12):e0296286. doi: 10.1371/journal.pone.0296286 (PMC10756540; doi:10.1371/journal.pone.0296286)

Supplementary Table 3- Identified haplogroups in *ND4* study data set

| Haplogroup ID | Network of the haplogroup | Haplotypes in the haplogroup |
| --- | --- | --- |
| **XV** | 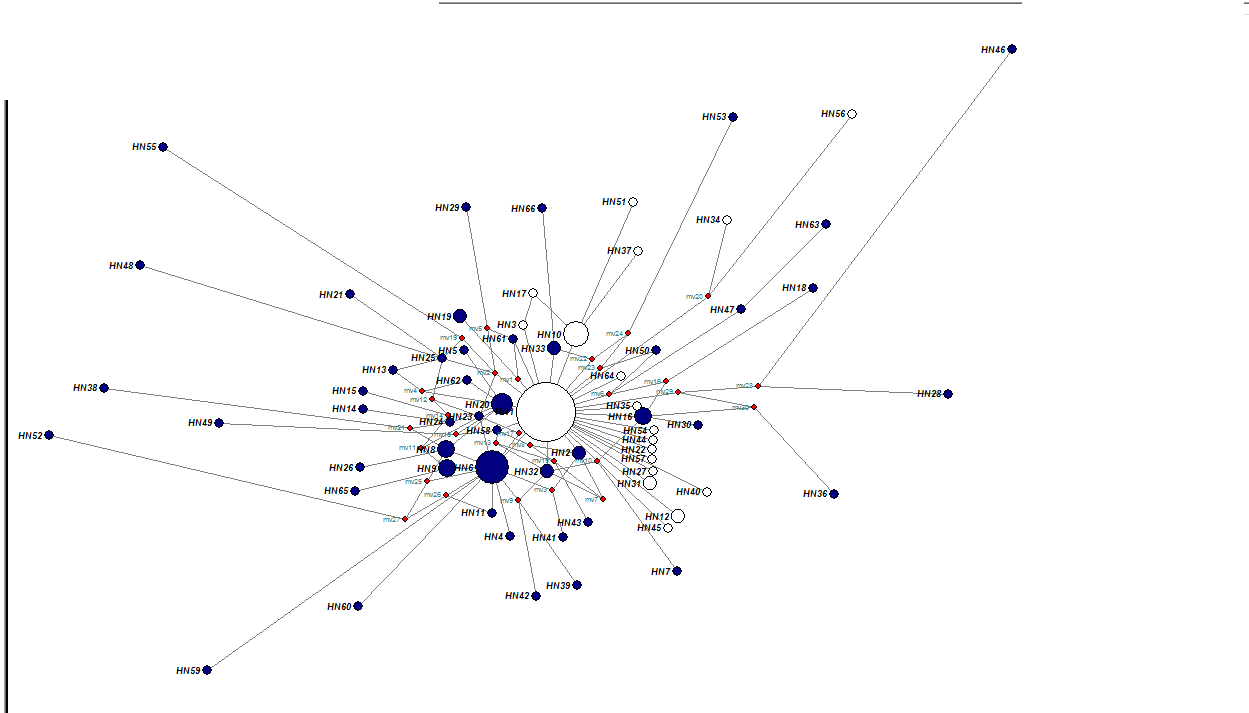 | ***HN1, HN2, HN4, HN5, HN6, HN7, HN8, HN9, HN11, HN13, HN14, HN15, HN16, HN18, HN19, HN20, HN21, HN23, HN24, HN25, HN26, HN28, HN29, HN30, HN32, HN33, HN36, HN38, HN39, HN41, HN42, HN43, HN46, HN47, HN48, HN49, HN50, HN52, HN53, HN55, HN58, HN59, HN60, HN61, HN62, HN63, HN65, HN66*** |
| **XVI** | **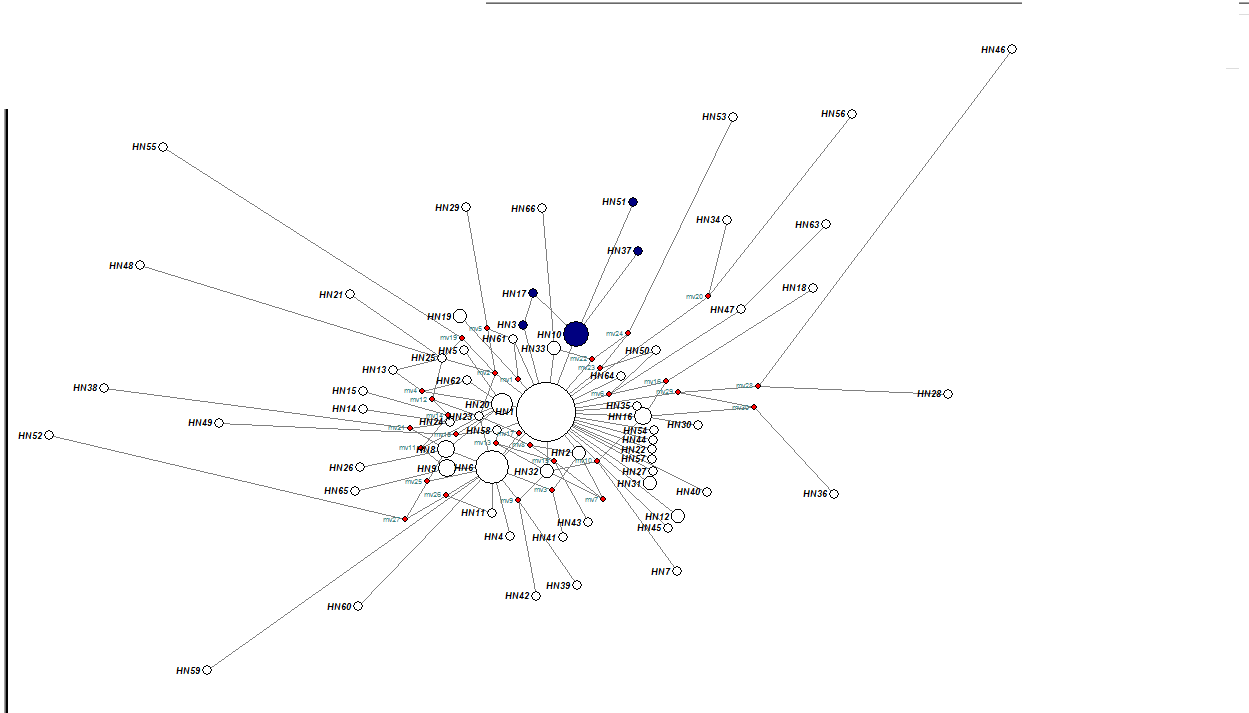** | ***HN3, HN10, HN17, HN37, HN51*** |
| **XVII** | **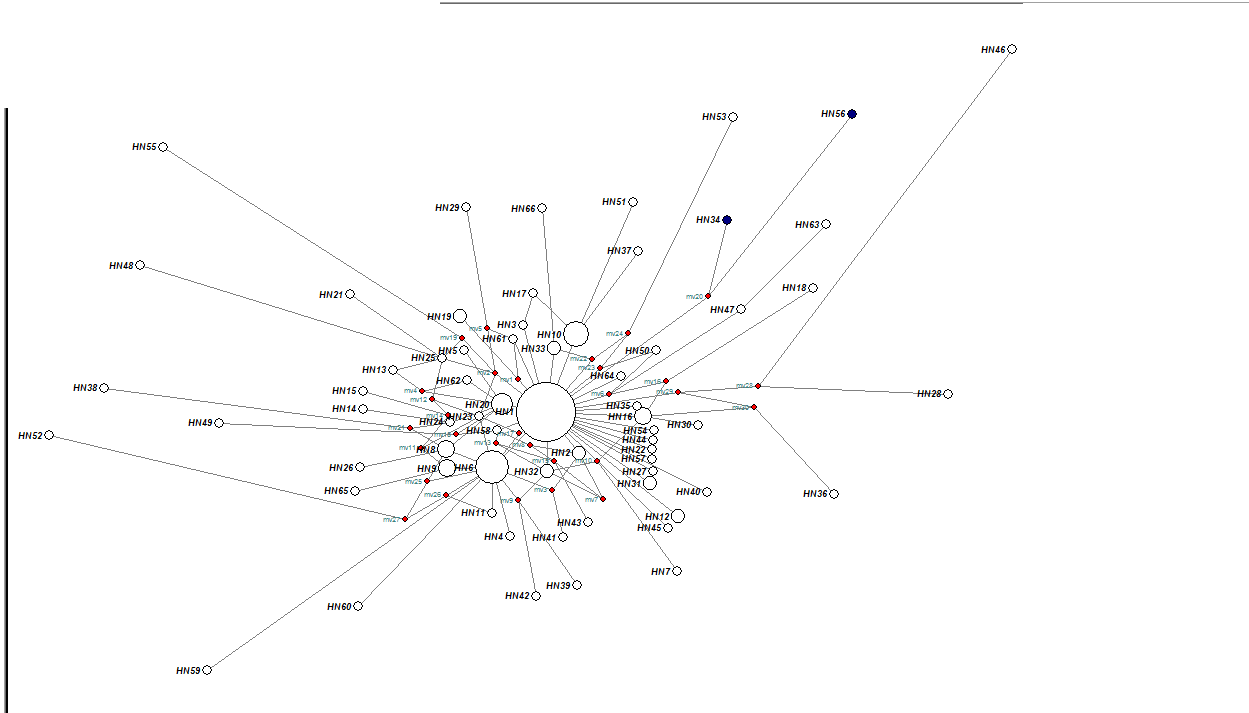** | ***HN34, HN56*** |


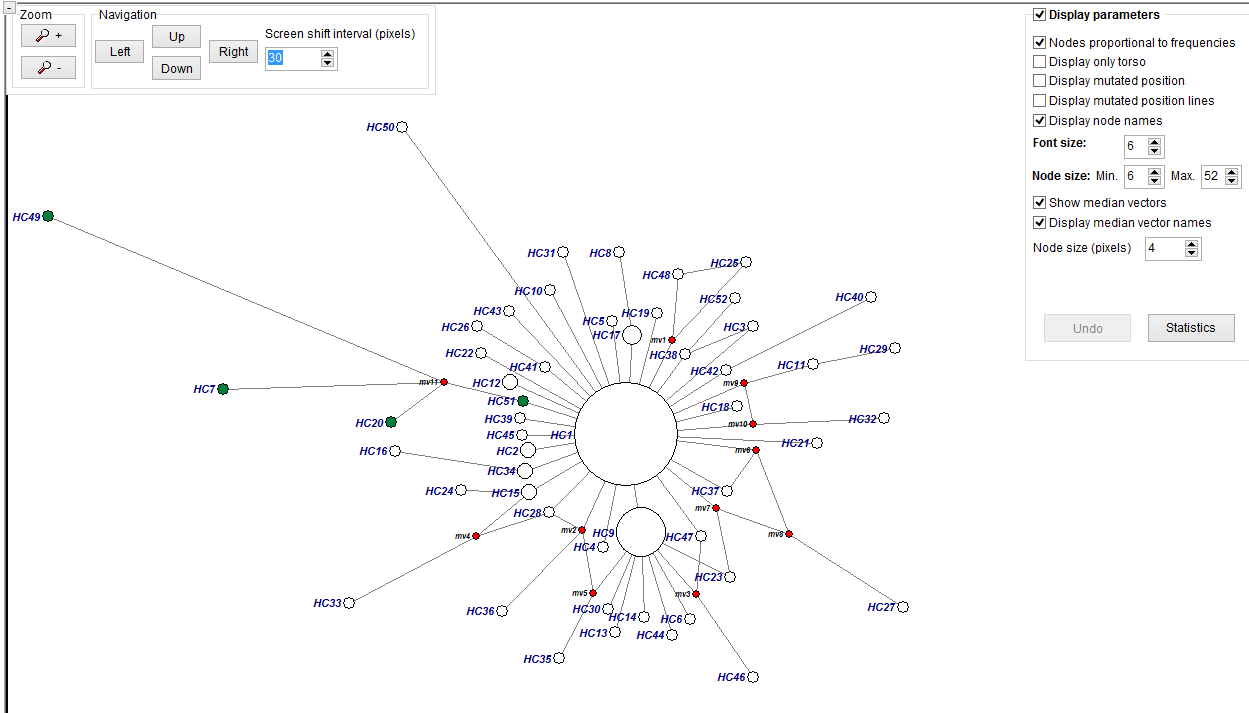

Supplement: S3 Table — Haplogroups identified from the ND4 study alignment of P. argentipes, along with corresponding haplotypes within each group. (DOC) [file pone.0296286.s003.doc]
