# Supplementary material for: Genetic diversity and phylogeography of Phlebotomus argentipes (Diptera: Psychodidae, Phlebotominae), using COI and ND4 mitochondrial gene sequences
Source: PLoS One. 2023 Dec 29;18(12):e0296286. doi: 10.1371/journal.pone.0296286 (PMC10756540; doi:10.1371/journal.pone.0296286)

Supplementary Table 4- Identified haplogroups in concatenated study data set

| Haplogroup ID | Network of the haplogroup | Haplotypes in the haplogroup |
| --- | --- | --- |
| **XVIII** | **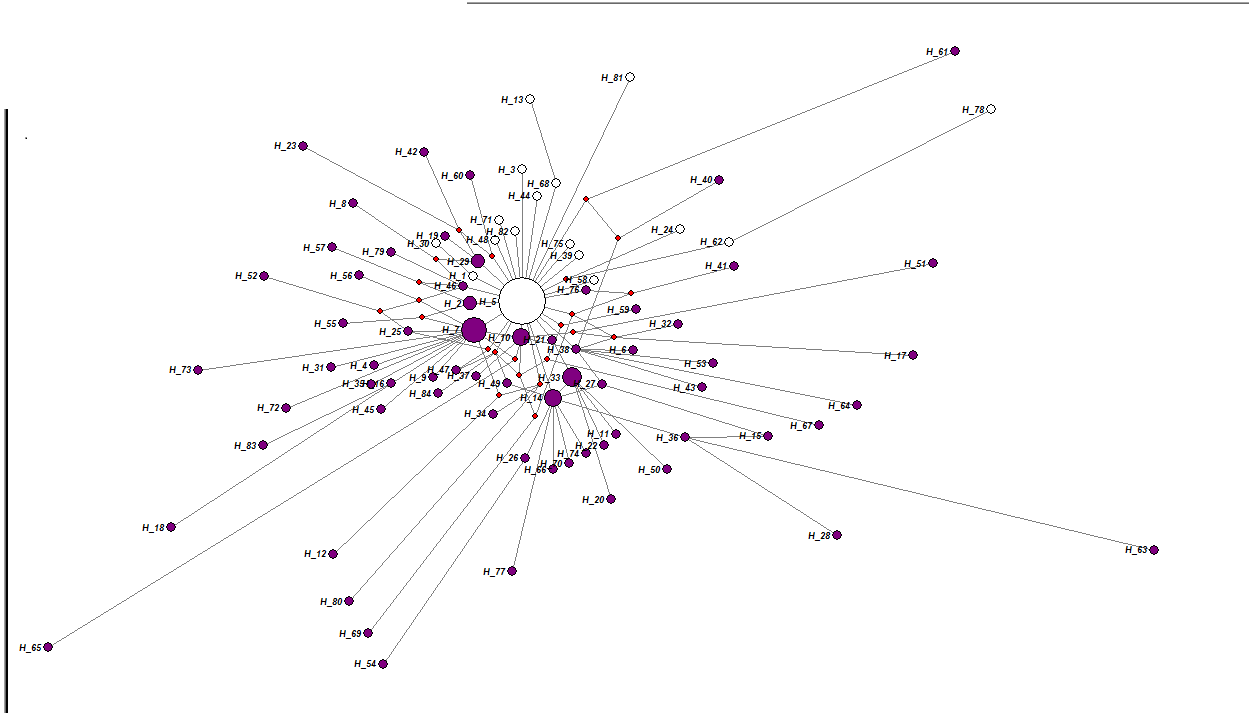** | ***H_2, H_4, H_5, H_6, H_7, H_8, H_9, H_10, H_11, H_12, H_14, H_15, H_16, H_17, H_18, H_19, H_20, H_21, H_22, H_23, H_25, H_26, H_27, H_28, H_29, H_31, H_32, H_33, H_34, H_35, H_36, H_37, H_38, H_40, H_41, H_42, H_43, H_45, H_46, H_47, H_49, H_50, H_51, H_52, H_53, H_54, H_55, H_56, H_57, H_59, H_60, H_61, H_63, H_64, H_65, H_66, H_67, H_69, H_70, H_72, H_73, H_74, H_76, H_77, H_79, H_80, H_83, H_84*** |
| **XIX** | 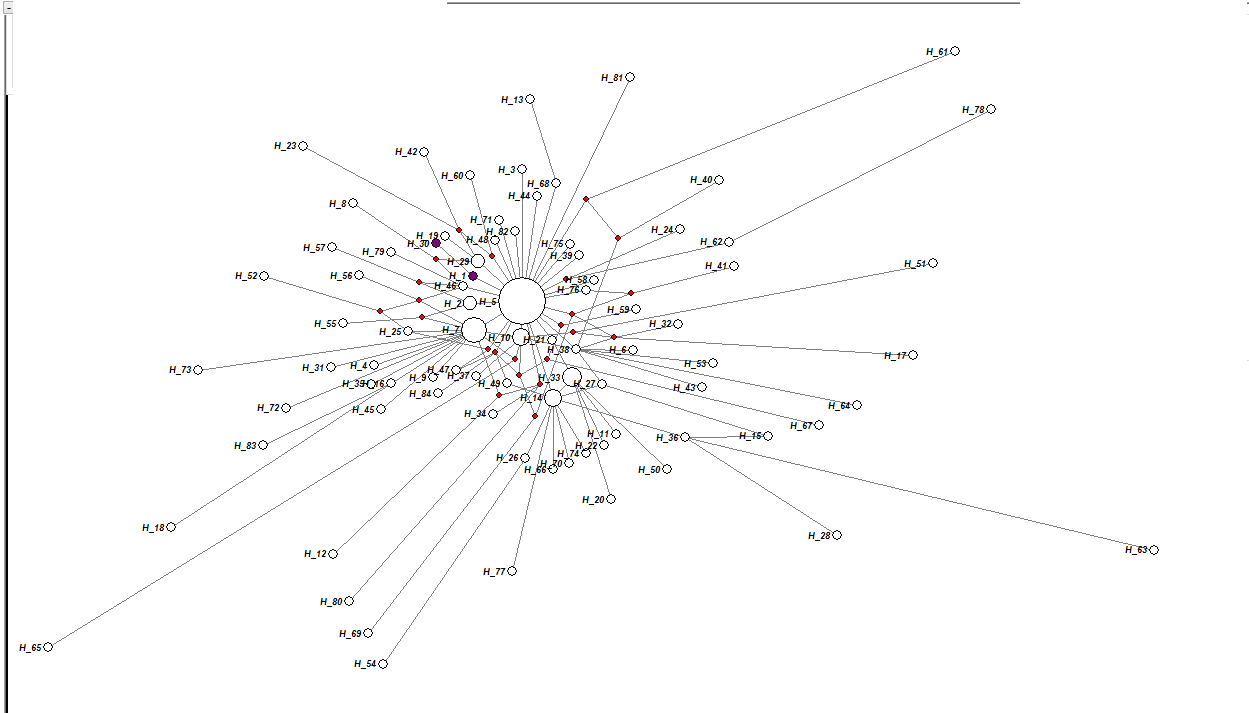 | ***H_1, H_30*** |
| **XX** | 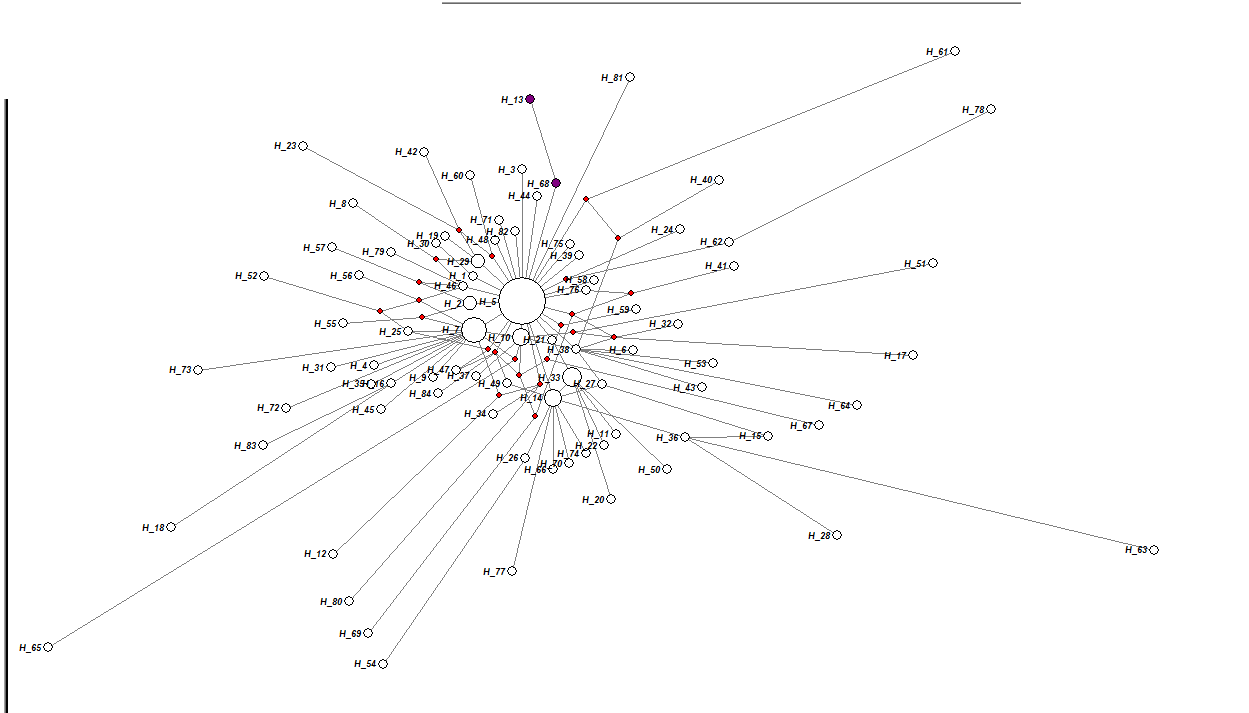 | ***H_13, H_68*** |
| **XXI** | 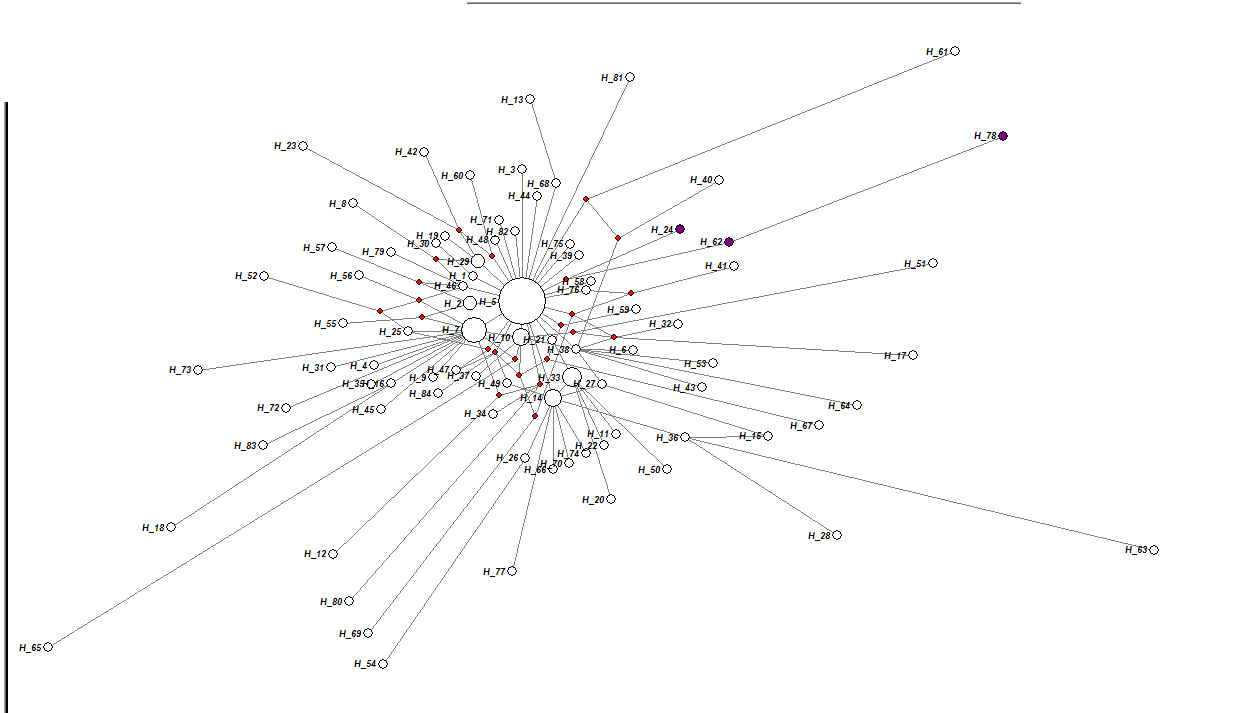 | ***H_24, H_62, H_78*** |


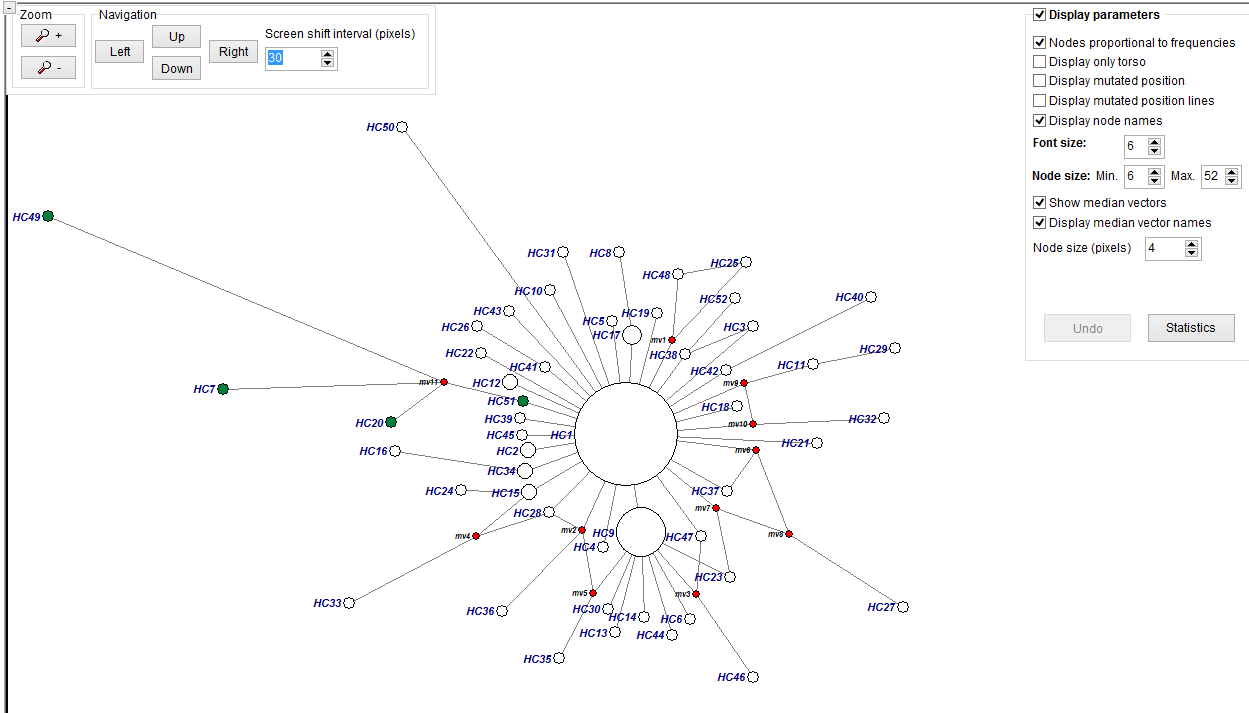

Supplement: S4 Table — Haplogroups identified from the concatenated alignment of P. argentipes, along with corresponding haplotypes within each group. (DOC) [file pone.0296286.s004.doc]
