# Supplementary material for: Genetic diversity and phylogeography of Phlebotomus argentipes (Diptera: Psychodidae, Phlebotominae), using COI and ND4 mitochondrial gene sequences
Source: PLoS One. 2023 Dec 29;18(12):e0296286. doi: 10.1371/journal.pone.0296286 (PMC10756540; doi:10.1371/journal.pone.0296286)

Supplementary Table 5- Identified haplogroups in *COI* regional data set

| Haplogroup ID | Network of the haplogroup | Haplotypes in the haplogroup |
| --- | --- | --- |
| **X** | **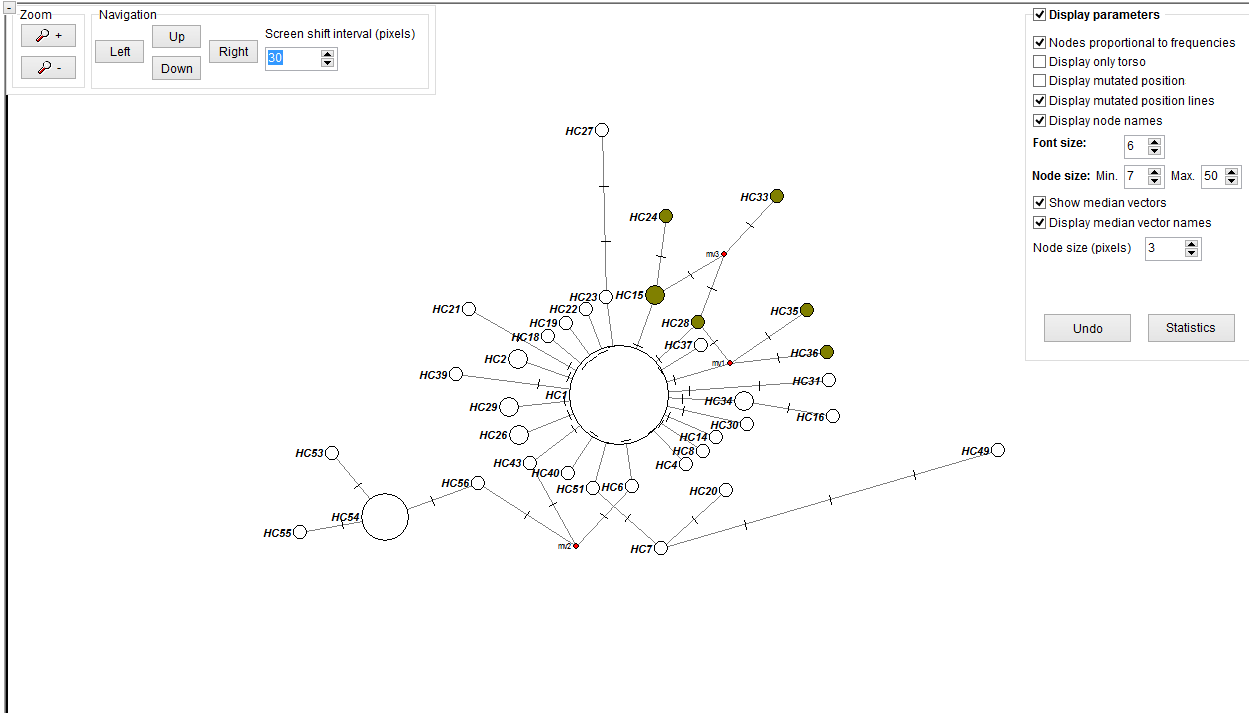** | ***HC15, HC24, HC28, HC33, HC35, HC36*** |
| **XI** | **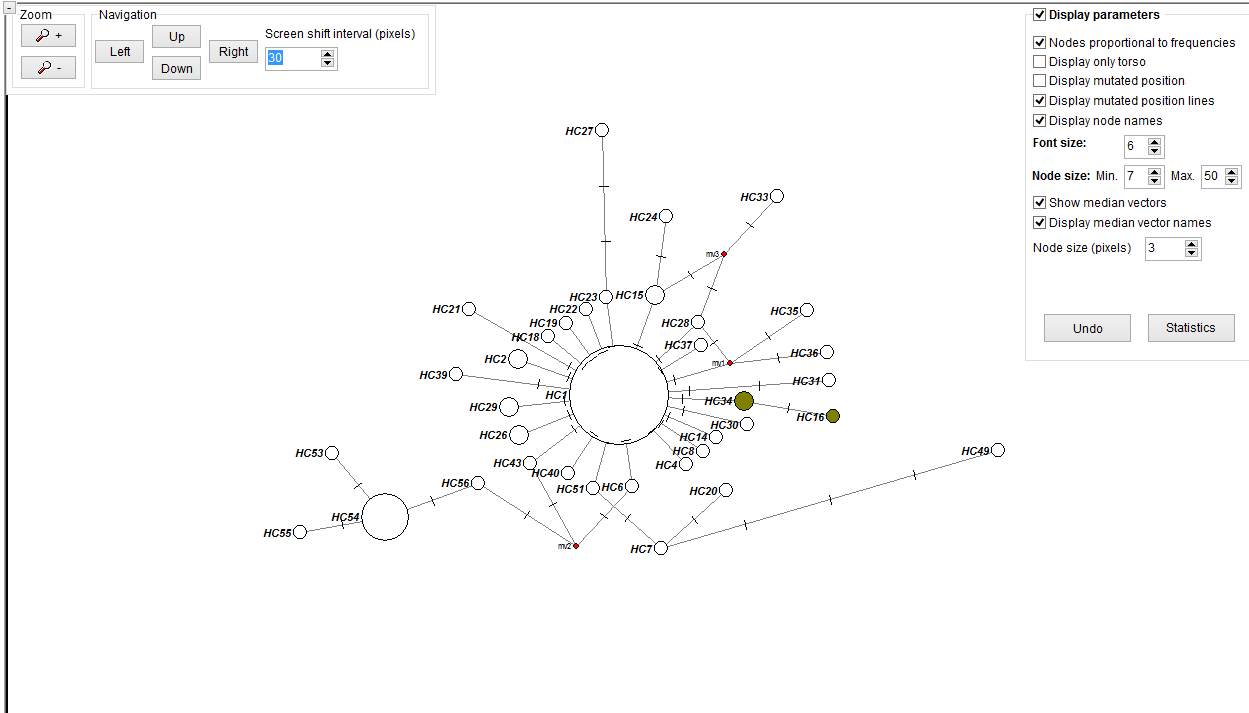** | ***HC16, HC34*** |
| **XII** | **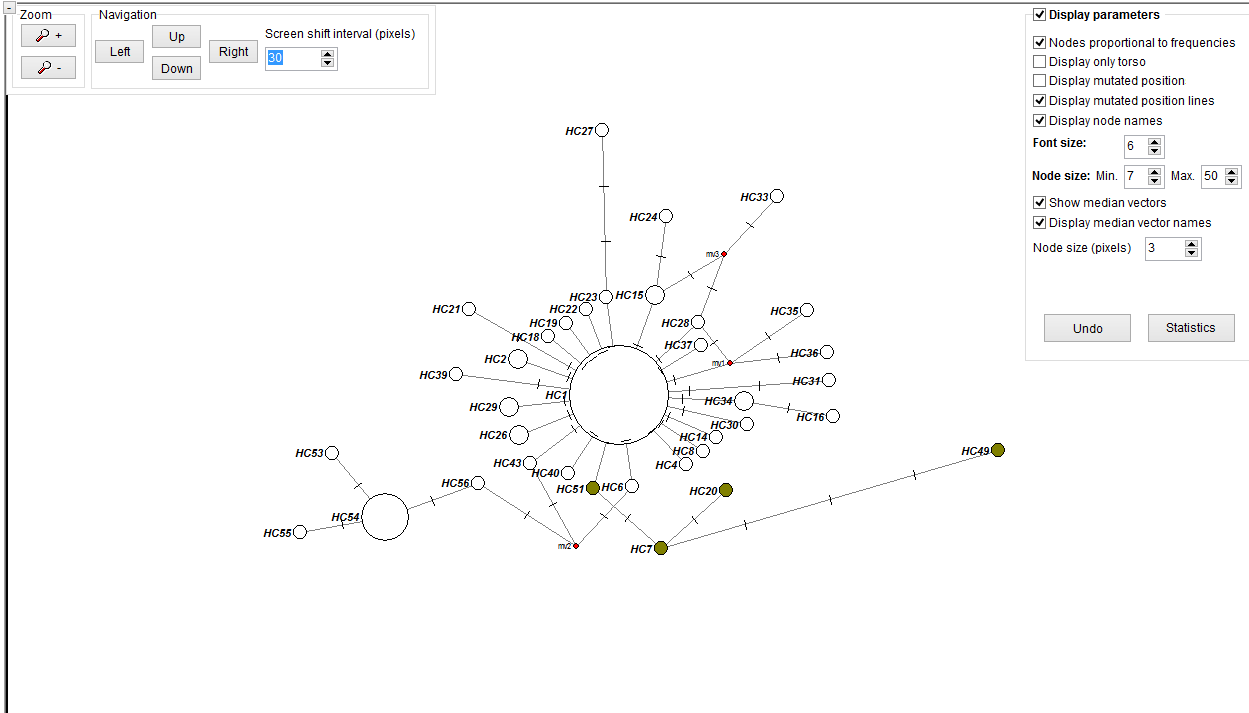** | ***HC7, HC20, HC49, HC51*** |
| **XIII** | **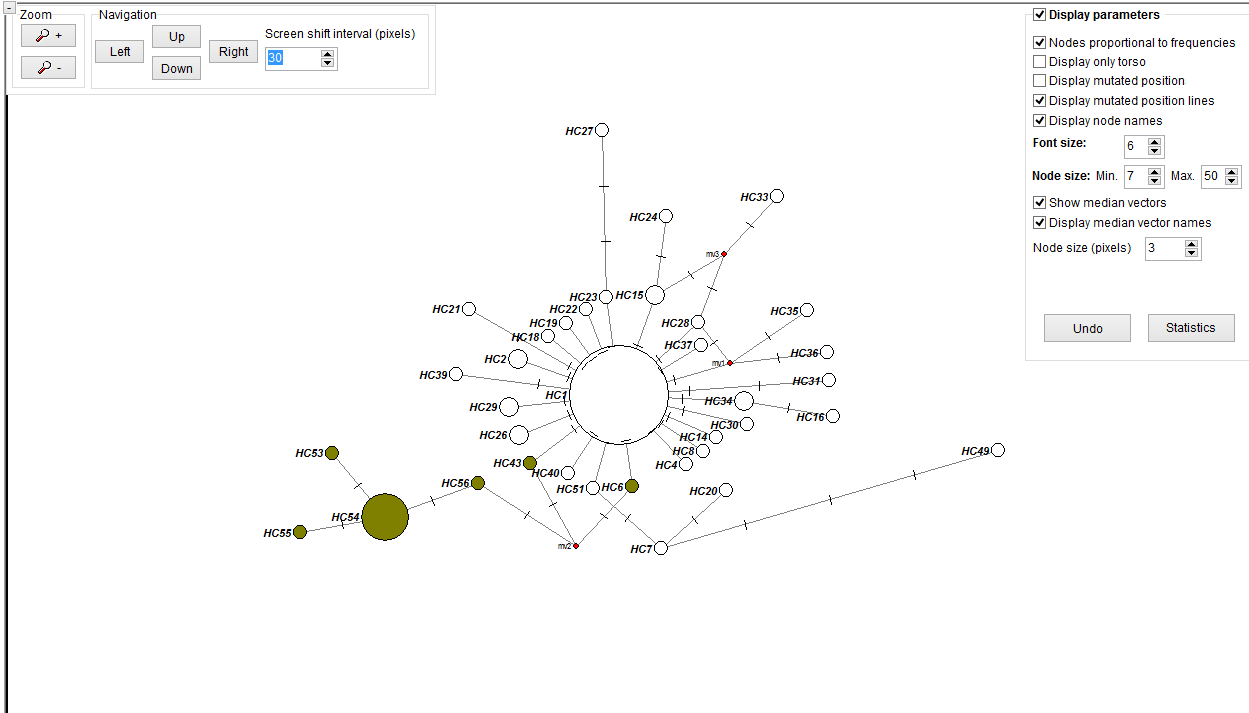** | ***HC6, HC43, HC53, HC54, HC55, HC56,*** |
| **XIV** | **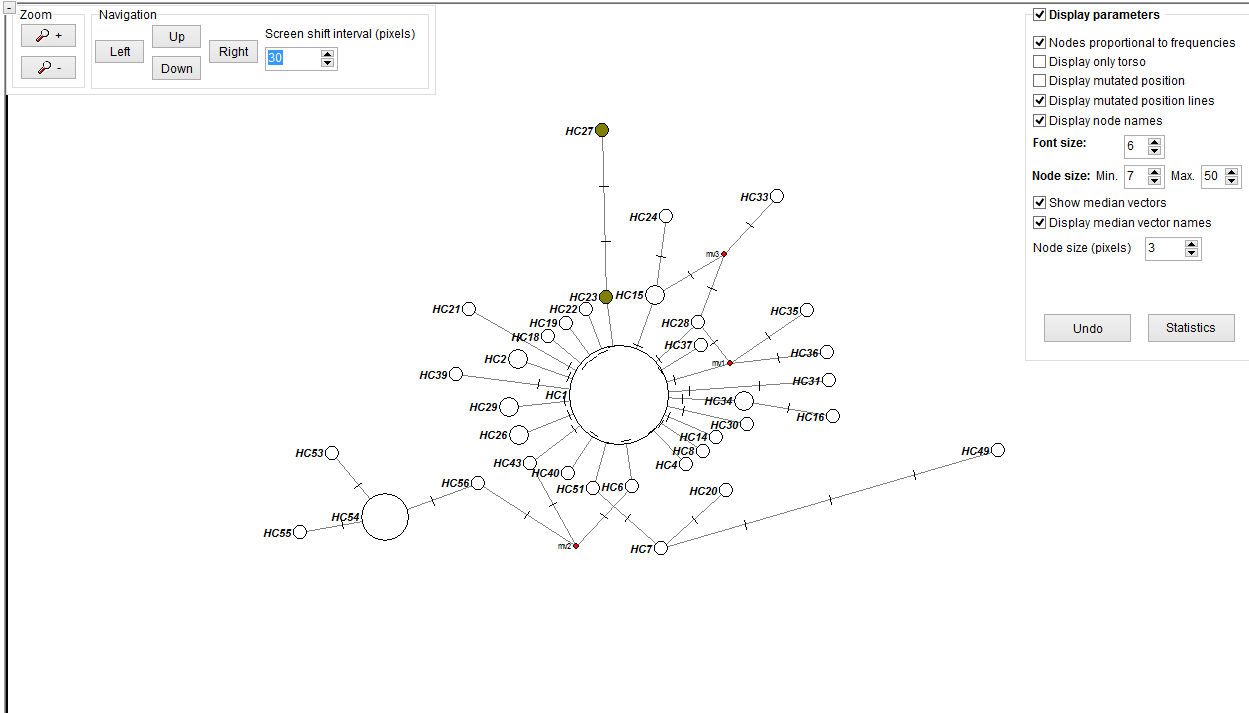** | ***HC23, HC27*** |


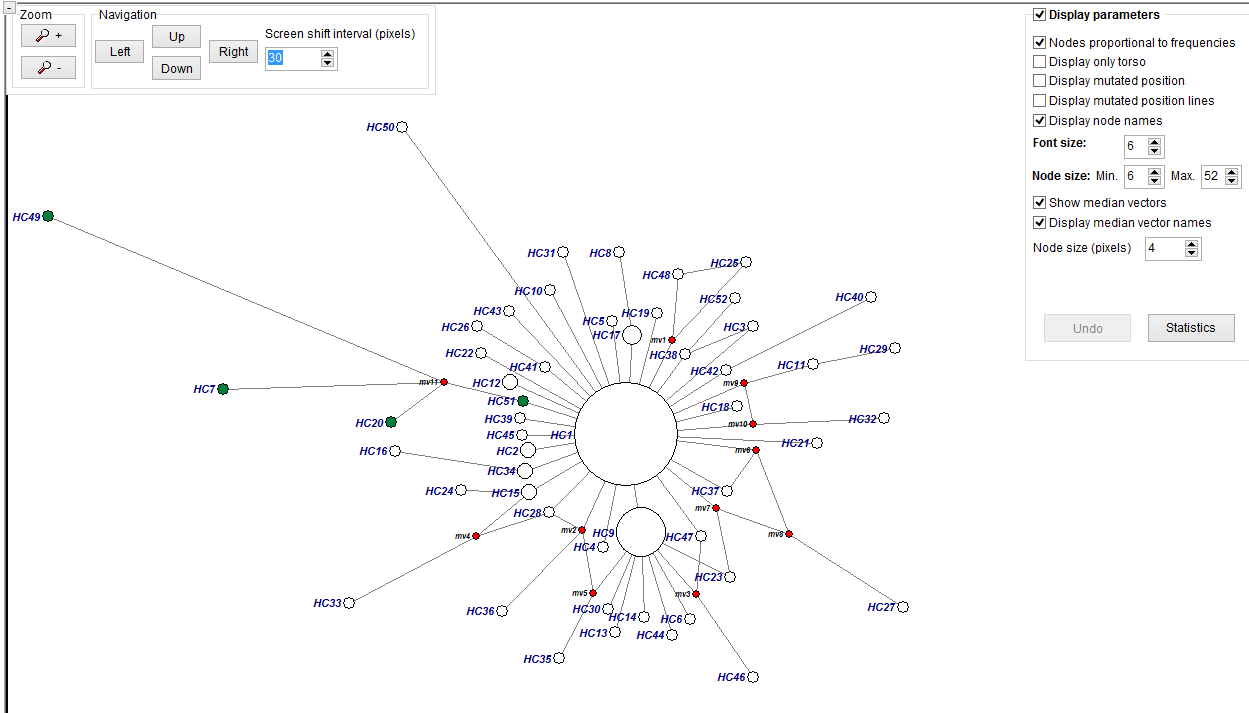

Supplement: S5 Table — Haplogroups identified from the COI regional alignment of P. argentipes, along with corresponding haplotypes within each group. (DOC) [file pone.0296286.s005.doc]
