# Supplementary material for: Genetic diversity and phylogeography of Phlebotomus argentipes (Diptera: Psychodidae, Phlebotominae), using COI and ND4 mitochondrial gene sequences
Source: PLoS One. 2023 Dec 29;18(12):e0296286. doi: 10.1371/journal.pone.0296286 (PMC10756540; doi:10.1371/journal.pone.0296286)
Supplement: S6 Table — Haplotypes presented in this table were identified from five sampling locations, each corresponding to four distinct alignments. The alignments include the COI study alignment (455 bp), COI regional alignment (343 bp), ND4 study alignment (598 bp), and concatenated alignment (1053 bp). The haplotypes represent unique genetic variants observed in each alignment and sampling location combination. (DOCX) [file pone.0296286.s006.docx]

**Table S6- Haplotypes generated through each sequence alignment and the relevant haplotype IDs of each population**

| **Study populations** | **Cytochrome Oxidase I** | | **NADH dehydrogenase subunit 4** | **Concatenated data set (*COI* + *ND4*)** |
| --- | --- | --- | --- | --- |
|  | ***COI* study alignment** | ***COI* regional alignment** |  |  |
| **Anuradhapura** | *HC1-HC12* | *HC1, HC2, HC4, HC6- HC8, HC14* | *HN1- HN17* | *H1- H22* |
| **Balangoda** | *HC1- HC2, HC9, HC15- HC25* | *HC1, HC2, HC15, HC16, HC18- HC24* | *HN1, HN6, HN8, HN10, HN16, HN18, HN19- HN30* | *H5, H7, H10, H1, H23- H43* |
| **Mirigama** | *HC1, HC9, HC15, HC17, HC26- HC30* | *HC1, HC15, HC26- HC30* | *HN1, HN2, HN6, HN20, HN31- HN38* | *H2, H5, H7,H14, H44- H51* |
| **Medirigiriya** | *HC1, HC9, HC12, HC17, HC31- HC37* | *HC1, HC31, HC37,* | *HN1, HN6, HN8, HN10, HN19, HN20, HN39- HN53* | *H5, H7, H10, H33, H52- H65* |
| **Hambantota** | *HC1, HC9, HC3, HC38- HC52* | *HC1, HC26, HC29, HC34, HC39, HC40, HC43, HC49, HC51* | *HN1, HN6, HN10, HN12, HN32, HN54- HN66* | *H5, H7, H33, H66- H84* |
| **India** | NA | *HC53- HC56* | NA | NA |
| **Sri Lanka** | NA | *HC1, HC54* | NA | NA |

(HC- *COI* haplotypes, HN- *ND4* haplotypes, H- concatenated haplotypes, NA- not applicable)
