# Supplementary figures and images for: Genetic diversity and phylogeography of Phlebotomus argentipes (Diptera: Psychodidae, Phlebotominae), using COI and ND4 mitochondrial gene sequences
Source: PLoS One. 2023 Dec 29;18(12):e0296286. doi: 10.1371/journal.pone.0296286 (PMC10756540; doi:10.1371/journal.pone.0296286)

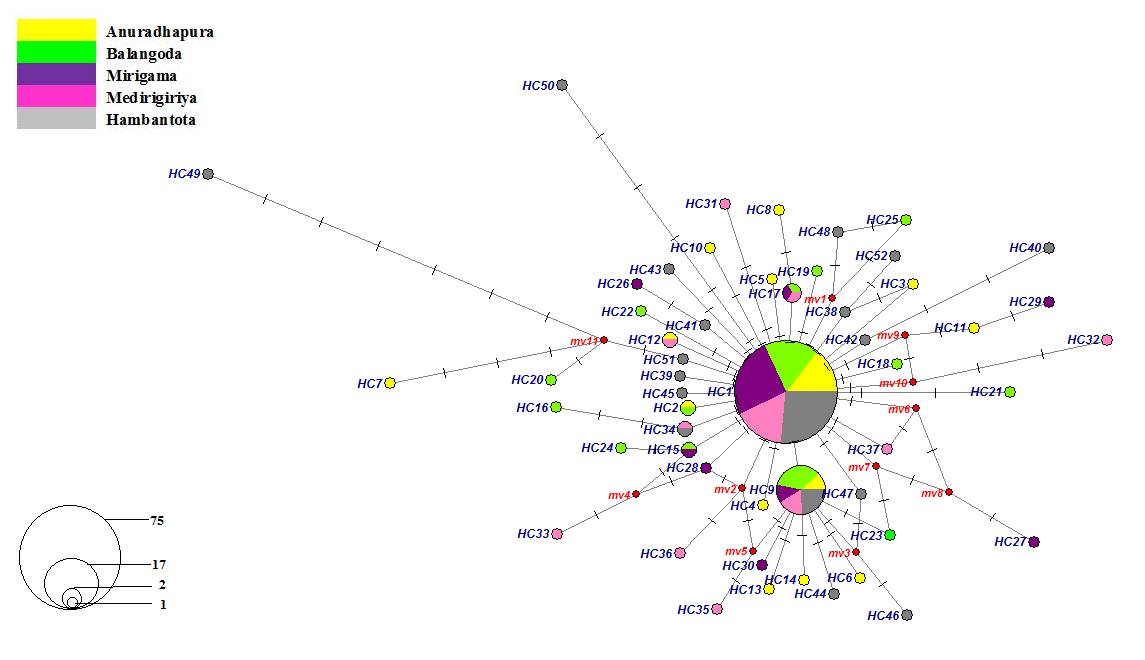

Supplement: S1 Fig — Circle size indicates the haplotype frequency, and the circle color the geographical location. Haplotype labels are written next to the corresponding circles, and the red circles indicate median vectors. (TIF) [file pone.0296286.s007.tif]

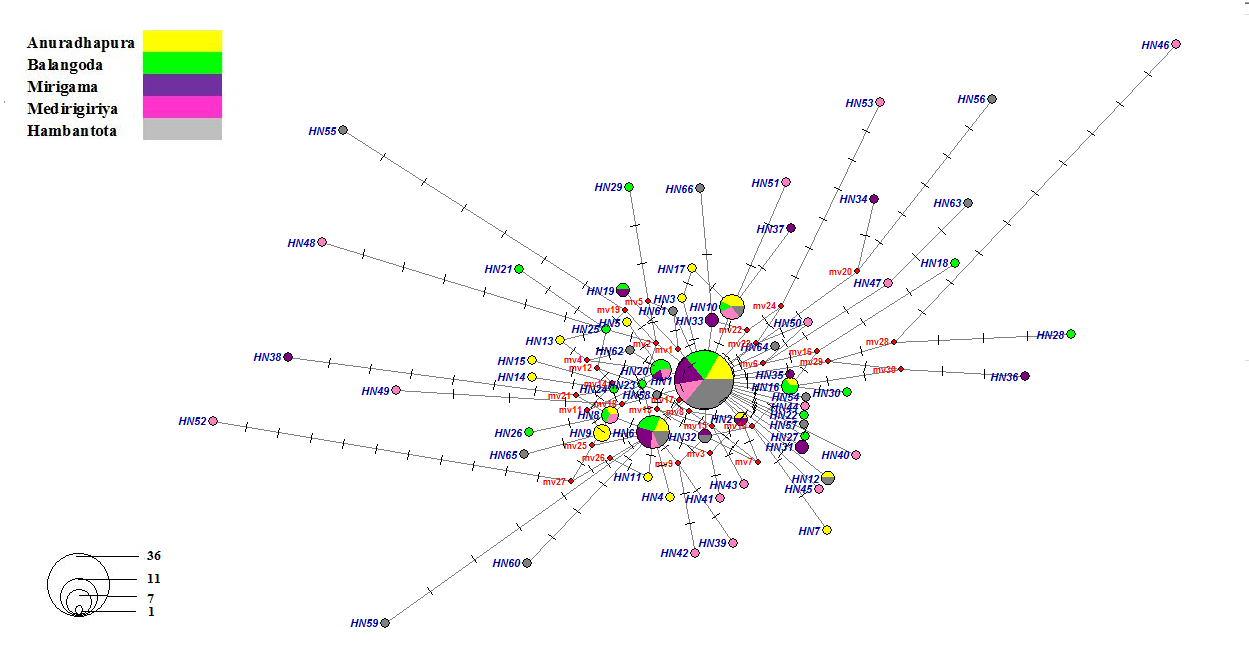

Supplement: S2 Fig — Circle size and color indicate the frequency and geographical location of the haplotypes respectively. Haplotype labels are written next to the corresponding circles, and the red circles indicate median vectors. (TIF) [file pone.0296286.s008.tif]
