# Supplementary material for: Genetic diversity and phylogeography of Phlebotomus argentipes (Diptera: Psychodidae, Phlebotominae), using COI and ND4 mitochondrial gene sequences
Source: PLoS One. 2023 Dec 29;18(12):e0296286. doi: 10.1371/journal.pone.0296286 (PMC10756540; doi:10.1371/journal.pone.0296286)
Supplement: Attachment — Submitted filename: PONE-D-23-20755_reviewer.pdf [file pone.0296286.s009.pdf]

PLOS ONE

Genetic diversity and phylogeography of Phlebotomus argentipes (Diptera: Psychodidae), using COI and ND4 mitochondrial gene sequences.

--Manuscript Draft--

**Manuscript Number:**	PONE-D-23-20755
**Article Type:**	Research Article
**Full Title:**	Genetic diversity and phylogeography of Phlebotomus argentipes (Diptera: Psychodidae), using COI and ND4 mitochondrial gene sequences.
**Short Title:**	Genetic diversity and phylogeography of Phlebotomus argentipes using COI and ND4 mitochondrial gene sequences.
**Corresponding Author:**	B.G.D.N.K. De Silva, Ph.D<br>University of Sri Jayewardenepura<br>Nugegoda, SRI LANKA
**Keywords:**	Leishmaniasis; Cutaneouse Leishmaniasis; SandFly; Phlebotomus argentipes; Genetic diversity; phylogeography; Cytochrome oxydase I; NADH dehydrogenase subunit; Mitochondrial gene; Sri Lanka; Haplotypes
**Abstract:**	Abstract<br>Background<br>Phlebotomus argentipes complex is the primary vector of Cutaneous Leishmaniasis, an upsurging health problem in recent Sri Lanka where vector control is crucial for proper disease management. Before implementing a successful vector control program, it is vital to understand the genetic diversity of the P. argentipes population in Sri Lanka. Several studies suggest that genetic divergence, caused by genetic drift or selection, influences the vector capacity and insecticide susceptibility of sandflies and other arthropod species. To develop novel control strategies for P. argentipes, the study of genetic diversity and phylogeography could provide insights into vector competence, insecticide resistance genes, and epidemiology. This study aimed to analyse the genetic diversity and phylogeography of the P. argentipes complex in Sri Lanka based on bioinformatics tools of two mitochondrial regions in modern representatives of P. argentipes populations.<br>Methodology<br>All P. argentipes individuals (n=159) were collected from five Cutaneous Leishmaniasis prevalence localities and identified. Two mitochondrial regions, Cytochrome Oxidase I (COI) and NADH dehydrogenase subunit 4 (ND4) were amplified using extracted DNA and sequenced. Partial sequences of the two mitochondrial genes were used in genetic diversity indices, phylogenetic and phylogeographic relationships analysis.<br>Principal Findings<br>Among five sampling localities, the highest genetic diversity for COI and ND4 was observed from Hambantota (Hd - 0.749, π - 0.00417) and Medirigiriya (Hd - 0.977, π - 0.01055), respectively. Phylogeographic analyses using COI sequences, including GenBank retrieved sequences, showed a superior divergence of P. argentipes haplotypes found in Sri Lanka. Results revealed that they have evolved from the Indian ancestral haplotype due to historical- geographical connections of the Indian subcontinent with Sri Lanka.<br>Conclusions<br>The findings describe the modern representatives of P. argentipes populations that derived from a common ancestor from the Indian sub-continent and it was confirmed by COI phylogeographic analysis. Furthermore, the present study revealed a high degree of polymorphism within the ND4 gene segment of P. argentipes, suggesting its utility as a parameter for estimating the genetic diversity of this species in Sri Lanka. This is the first study on the genetic diversity analysis of P. argentipes using the ND4 marker which gave a precious image of divergence with high polymorphism.
**Order of Authors:**	B.G.D.N.K De Silva, PhD (USJP), BSc (USJP)
	W. Methsala Madurangi Wedage, BSc (USJP)
	Iresha N Harischandra, PhD (USJP), BSc (UOC)

Powered by Editorial Manager® and ProduXion Manager® from Aries Systems Corporation

	O.V.D.S Jagathpriya Weerasena, PhD (UOC), BSc (UOC)
**Additional Information:**	
**Question**	**Response**
**Financial Disclosure**<br><br>Enter a financial disclosure statement that describes the sources of funding for the work included in this submission. Review the submission guidelines for detailed requirements. View published research articles from *PLOS ONE* for specific examples.<br><br>This statement is required for submission and **will appear in the published article** if the submission is accepted. Please make sure it is accurate.<br><br>**Unfunded studies**<br>Enter: *The author(s) received no specific funding for this work.*<br><br>**Funded studies**<br>Enter a statement with the following details:<br>• Initials of the authors who received each award<br>• Grant numbers awarded to each author<br>• The full name of each funder<br>• URL of each funder website<br>• Did the sponsors or funders play any role in the study design, data collection and analysis, decision to publish, or preparation of the manuscript?<br>• **NO** - Include this sentence at the end of your statement: *The funders had no role in study design, data collection and analysis, decision to publish, or preparation of the manuscript.*<br>• **YES** - Specify the role(s) played.<br><br>* typeset	This research was funded by the University Research Grant, University of Sri Jayewardenepura, grant number ASP/01/RE/SCI/2017/53, and the Centre for Biotechnology, Department of Zoology, Faculty of Applied Sciences, University of Sri Jayewardenepura, Sri Lanka. Snr. Prof. B.G.D.N.K. De Silva received the funding. The funders had no role in study design, data collection, analysis, the decision to publish, or manuscript preparation.
**Competing Interests**<br><br>Use the instructions below to enter a competing interest statement for this submission. On behalf of all authors, disclose any competing interests that could be perceived to bias this work—acknowledging all financial support and any other relevant financial or non-financial competing interests.	The authors have declared that no competing interests exist

This statement is required for submission and **will appear in the published article** if the submission is accepted. Please make sure it is accurate and that any funding sources listed in your Funding Information later in the submission form are also declared in your Financial Disclosure statement.<br><br>View published research articles from *PLOS ONE* for specific examples.<br><br>**NO authors have competing interests**<br><br>Enter: *The authors have declared that no competing interests exist.*<br><br>**Authors with competing interests**<br><br>Enter competing interest details beginning with this statement:<br><br>*I have read the journal's policy and the authors of this manuscript have the following competing interests: [insert competing interests here]*<br><br>* typeset	
**Ethics Statement**<br><br>Enter an ethics statement for this submission. This statement is required if the study involved:<br><br>• Human participants<br>• Human specimens or tissue<br>• Vertebrate animals or cephalopods<br>• Vertebrate embryos or tissues<br>• Field research<br><br>Write "N/A" if the submission does not require an ethics statement.<br><br>General guidance is provided below. Consult the submission guidelines for detailed instructions. **Make sure that all**	N/A

**information entered here is included in the Methods section of the manuscript.**<br><br>**Format for specific study types**<br><br>**Human Subject Research (involving human participants and/or tissue)**<br>• Give the name of the institutional review board or ethics committee that approved the study<br>• Include the approval number and/or a statement indicating approval of this research<br>• Indicate the form of consent obtained (written/oral) or the reason that consent was not obtained (e.g. the data were analyzed anonymously)<br><br>**Animal Research (involving vertebrate animals, embryos or tissues)**<br>• Provide the name of the Institutional Animal Care and Use Committee (IACUC) or other relevant ethics board that reviewed the study protocol, and indicate whether they approved this research or granted a formal waiver of ethical approval<br>• Include an approval number if one was obtained<br>• If the study involved *non-human primates*, add *additional details* about animal welfare and steps taken to ameliorate suffering<br>• If anesthesia, euthanasia, or any kind of animal sacrifice is part of the study, include briefly which substances and/or methods were applied<br><br>**Field Research**<br><br>Include the following details if this study involves the collection of plant, animal, or other materials from a natural setting:<br>• Field permit number<br>• Name of the institution or relevant body that granted permission	
**Data Availability**<br><br>Authors are required to make all data underlying the findings described fully available, without restriction, and from the time of publication. PLOS allows rare exceptions to address legal and ethical	Yes - all data are fully available without restriction

concerns. See the PLOS Data Policy and FAQ for detailed information.<br><br>A Data Availability Statement describing where the data can be found is required at submission. Your answers to this question constitute the Data Availability Statement and **will be published in the article**, if accepted.<br><br>**Important:** Stating 'data available on request from the author' is not sufficient. If your data are only available upon request, select 'No' for the first question and explain your exceptional situation in the text box.<br><br>Do the authors confirm that all data underlying the findings described in their manuscript are fully available without restriction?	
**Describe where the data may be found in full sentences. If you are copying our sample text, replace any instances of XXX with the appropriate details.**<br><br>• If the data are **held or will be held in a public repository**, include URLs, accession numbers or DOIs. If this information will only be available after acceptance, indicate this by ticking the box below. For example: *All XXX files are available from the XXX database (accession number(s) XXX, XXX.*).<br>• If the data are all contained **within the manuscript and/or Supporting Information files**, enter the following: *All relevant data are within the manuscript and its Supporting Information files.*<br>• If neither of these applies but you are able to provide **details of access elsewhere**, with or without limitations, please do so. For example:<br><br>*Data cannot be shared publicly because of [XXX]. Data are available from the XXX Institutional Data Access / Ethics Committee (contact via XXX) for researchers who meet the criteria for access to confidential data.*	All relevant data are within the manuscript and its Supporting Information files.

The data underlying the results presented in the study are available from (include the name of the third party and contact information or URL).<br>• This text is appropriate if the data are owned by a third party and authors do not have permission to share the data.<br><br>* typeset	
Additional data availability information:	Tick here if the URLs/accession numbers/DOIs will be available only after acceptance of the manuscript for publication so that we can ensure their inclusion before publication.

Powered by Editorial Manager® and ProduXion Manager® from Aries Systems Corporation

Manuscript Click here to access/download;Manuscript;Manuscript.doc

Genetic diversity and phylogeography of *Phlebotomus argentipes* (Diptera: Psychodidae), using *COI* and *ND4* mitochondrial gene sequences.

Wedage Methsala Madurangi Wedage[1], Iresha Nilmini Harischandra[2], O. V. D. S. Jagathpriya Weerasena[3], B. G. D. Nissanka K. De Silva[*1,4]

[1] Center for Biotechnology, Department of Zoology, Faculty of Applied Sciences, University of Sri Jayewardenepura, Nugegoda 10250, Sri Lanka;

[2] Vidya Sethu Foundation, 36/1B, Heenatikumbura Road, Thalangama North, Battaramulla, 10120, Sri Lanka;

[3] Institute of Biochemistry, Molecular Biology and Biotechnology (IBMBB), University of Colombo, Kumaratunga Munidasa Mawatha, Colombo 03, Sri Lanka;

[4] Genetics and Molecular Biology Unit, Faculty of Applied Sciences, University of Sri Jayewardenepura, Nugegoda 10250, Sri Lanka;

[*] Corresponding Author- B. G. D. Nissanka K. De Silva (ORCID- 0000-0001-8725-4393)

E-mail: nissanka@sci.sjp.ac.lk

Author Contributions

Conceptualization: B.G.D. Nissanka K. de Silva, Iresha Nilmini Harischandra, Wedage Methsala Madurangi Wedage

Data curation: Wedage Methsala Madurangi Wedage

Formal analysis: Wedage Methsala Madurangi Wedage, Iresha Nilmini Harischandra

Funding acquisition: B. G. D. Nissanka K. de Silva

Investigation: Wedage Methsala Madurangi Wedage, Iresha Nilmini Harischandra

Methodology: Wedage Methsala Madurangi Wedage, Iresha Nilmini Harischandra, B. G. D. Nissanka K. de Silva

Project administration: B. G. D. Nissanka K. de Silva, Wedage Methsala Madurangi Wedage

Resources: B. G. D. Nissanka K. de Silva

Software: Wedage Methsala Madurangi Wedage

Supervision: B. G. D. Nissanka K. de Silva, Iresha Nilmini Harischandra, O. V. D. S. Jagathpriya Weerasena

Validation: Wedage Methsala Madurangi Wedage

Writing – original draft: Wedage Methsala Madurangi Wedage

Writing – review & editing: B. G. D. Nissanka K. de Silva, Iresha Nilmini Harischandra, O. V. D. S. Jagathpriya Weerasena

Abstract

Background

Phlebotomus argentipes complex is the primary vector of ~~Cutaneous Leishmaniasis~~, an upsurging health problem in recent Sri Lanka where vector control is crucial for proper disease management. Before implementing a successful vector control program, it is vital to understand the genetic diversity of the *P. argentipes* population in Sri Lanka. Several studies suggest that genetic divergence, caused by genetic drift or selection, influences the vector capacity and insecticide susceptibility of sandflies and other arthropod species. To develop novel control strategies for *P. argentipes*, the study of genetic diversity and phylogeography could provide insights into vector competence, insecticide resistance genes, and epidemiology. This study aimed to analyse the genetic diversity and phylogeography of the *P. argentipes* complex in Sri Lanka based on bioinformatics tools of two mitochondrial regions in modern representatives of *P. argentipes* populations.

Methodology

All *P. argentipes* individuals (n=159) were collected from five Cutaneous Leishmaniasis prevalence localities and identified. Two mitochondrial regions, ~~Cytochrome Oxidase I~~ (*COI*) and NADH dehydrogenase subunit 4 (*ND4*) were amplified using extracted DNA and sequenced. Partial sequences of the two mitochondrial genes were used in genetic diversity indices, phylogenetic and phylogeographic relationships analysis.

Principal Findings

Among five sampling localities, the highest genetic diversity for *COI* and *ND4* was observed from Hambantota (Hd - 0.749, π - 0.00417) and Medirigiriya (Hd - 0.977, π - 0.01055), respectively. Phylogeographic analyses using *COI* sequences, including GenBank retrieved

sequences, showed a superior divergence of *P. argentipes* haplotypes found in Sri Lanka. Results revealed that they have evolved from the Indian ancestral haplotype due to historical-geographical connections of the Indian subcontinent with Sri Lanka.

Conclusions

The findings describe the modern representatives of *P. argentipes* populations that derived from a common ancestor from the Indian sub-continent and it was confirmed by *COI* phylogeographic analysis. Furthermore, the present study revealed a high degree of polymorphism within the *ND4* gene segment of *P. argentipes*, suggesting its utility as a parameter for estimating the genetic diversity of this species in Sri Lanka. This is the first study on the genetic diversity analysis of *P. argentipes* using the *ND4* marker which gave a precious image of divergence with high polymorphism.

Author Summary

Recent studies in Sri Lanka indicate a localised distribution of leishmaniasis transmission. The *P. argentipes* complex is the vector of Cutaneous Leishmaniasis (CL) and Visceral Leishmaniasis (VL) transmission in the Indian subcontinent and Sri Lanka. This study aimed to analyse the genetic diversity of *P. argentipes* in Sri Lanka by using *COI* and *ND4* two mitochondrial markers, and phylogeographic analysis in Southern India using only the *COI* marker. In addition, a concatenated phylogenetic analysis was carried out for the first time for this species to detect the relationship of divergence. Furthermore, regional *COI* alignment differentiated five haplogroups suggesting their clear divergence from the Indian ancestral haplogroup representing South India and the Northern part of Sri Lanka.

Introduction

Genetic diversity is one of the fundamental elements of evolution and the foundation material that adaptation and speciation depend on [1]. In general, most of the common vector species have large populations and high levels of genetic diversity due to their gene flow or outbreeding enhancement (heterosis), which is a kind of mechanism that imparts high fitness in natural vector populations [2]. However, the existence of higher genetic diversity in a vector population can lead to the failure of some approaches of vector control and disease management practices as the incidence of building up in resistance to insecticides and sterile males [2, 3].

Out of 800 sandfly species in the world *Phlebotomus argentipes* Annandale and Brunetti is one of the ~~major~~ vectors for Visceral Leishmaniasis (VL) and Cutaneous Leishmaniasis (CL). Cutaneous Leishmaniasis is known as the most common form, annually reporting 0.7-1.2 million cases worldwide [4] and caused by nearly 20 species of obligate intracellular protozoa *Leishmania sp*. (Euglenozoa: Trypanosomatidae) [5]. The epidemiology of leishmaniasis has got attention in Sri Lanka since 2001 as a noticeable, non-communicable disease [6]. Around 1,991 clinically confirmed annual CL cases have been recorded and were mainly endemic in North Central Province (Anuradhapura District and Polonnaruwa District) and Southern Province (Matara and Hambantota districts) in the country (Figure 1) [7]. Therefore, recent studies on leishmaniasis epidemiology have shown the augmentation of disease control due to a need for proper disease management practices.

The distribution, abundance and diversity of the phlebotomine sandflies are believed to be high in the country and associated with the factors such as altitude, the distance between populations, habitat modifications, anthropogenic landscape fragmentation, vegetation types, geographic barriers, host communities, host species turnover, vectorial capacity and insecticide susceptibility [8- 10].

Figure 1- The distribution of Cutaneous Leishmaniasis incidence in each district, Sri Lanka 2021 (Weekly records 2020 published by Epidemiology Unit, Ministry of Health, Sri Lanka.

Molecular markers, including genomic DNA markers (microsatellites, ITS2) and mitochondrial markers (Cyt *b*, *COI*, and *ND4*) have been used [11-17] to determine the genetic diversity and phylogeography of different sandfly species (*P. papatasi, P. perniciosus* and *Lu. Longipalpis*). Compared to nuclear DNA, maternally inherited mitochondrial genes reflect evolutionary history more accurately due to their clonal inheritance, lack of recombination, and higher mutation rate [18, 19]. Thus, mitochondrial genes have been used in evolutionary studies of vector species including phlebotomine sandflies. However, to estimate the precise image of the phylogeography of sandfly species, most of the studies had been used a few mitochondrial genes rather than a single mitochondrial gene [20-25]. In the present study, extended fragments of the cytochrome oxidase I (*COI*) gene, exceeding the lengths employed in previous investigations of *P. argentipes*, and NADH dehydrogenase subunit 4 (*ND4*) fragments characterized by elevated mutation rates, were utilized to explore the phylogeography and genetic diversity of *P. argentipes* in Sri Lanka.

Despite being the primary vector for cutaneous leishmaniasis (CL) in Sri Lanka, the existing scientific knowledge pertaining to the taxonomy, genetic variability, vectorial capacity, xeno-diagnoses, insecticide resistance, and bionomics of *P. argentipes* remains insufficient. This knowledge gap poses limitations on the development of effective vector control programs in the future. To address this, the present study employs molecular phylogenetic analysis, utilizing two mitochondrial DNA (mtDNA) gene fragments from modern representatives of *P. argentipes*, along with available historical data on the physical geography of Sri Lanka and India. The study aims to provide a phylogeographical interpretation of *P. argentipes*, enhancing the understanding of its genetic structure and distribution. This analysis provides a coherent

explanation of historical, geographical and behavioral factors responsible for the genetic diversity of extant *P. argentipes* populations in Sri Lanka.

Methods

Study area, sample collection, and identification

Phlebotomus argentipes adult flies were collected (n=159) using randomly placed CDC light traps [26], sticky traps [27], and cattle-baited net traps [28] from five localities in Sri Lanka from March 2018 to March 2020. The sampling localities were selected based on human CL disease prevalence in the country as per the reported patient numbers [7] (Figure 2, Table 1). Multiple collections at each site were conducted. The samples were stored in 70% ethanol at the field and immediately transferred to the -80 ^{0}C freezer at the Molecular Genetics Laboratory of the University of Sri Jayewardenepura for further analysis. *P. argentipes* individuals were identified using standard keys of Lewis (1978) [29] and Lane (1993) [30] prior to extract DNA.

Table 1- GPS coordinates and the number of *Phlebotomus argentipes* samples from each site.

**Location ID**	**Code**	**GPS coordinates (WGS 84)**		**Number of *P. argentipes* specimens**
		**Longitude**	**Latitude**	
**Hambantota**	HAM	6.313056°	81.224167°	41
**Mirigama**	MIR	7.258331°	80.150831°	31
**Medirigiriya**	MED	8.143417°	81.039300°	28
**Balangoda**	BAL	6.671942°	81.023608°	32
**Anuradhapura**	ANU	8.239167°	80.500000°	27

WGS84- World Geodetic System 1984

Figure 2- Geographic distribution of *Phlebotomus argentipes* samples analysed

Sampling localities of *P. argentipes* specimens collected (triangles) and GenBank sequences retrieved for mitochondrial DNA fragments from Sri Lanka (square), and those from India (circle).

DNA extraction, PCR amplification and sequencing

According to the manufacturers' instructions, genomic DNA was extracted from *P. argentipes* individual sandflies without head regions, using DNeasy Blood & Tissue kit (Qiagen, California, USA). The head parts were preserved with 70% ethanol for further experiments. The *COI* region was partially amplified [31] in 25 µL, containing 1X PCR buffer, 1.5 mM $MgCl_2$, 0.2 mM dNTPs, 0.5 µM of each primer, 1U of *Taq* DNA polymerase (Promega, USA) and 1-2 ng of template DNA. The cyclic conditions [31] were modified with an additional step of 94 °C for 5 min as DNA preheat. The *ND4* region was amplified [14] in a reaction volume of 25 µL contained 1X PCR buffer, 1.5 mM $MgCl_2$, 0.2 mM dNTPs, 0.5 µM of each primer, and 1-2 ng of template DNA. Cyclic conditions were: 95 °C for 5 min, followed by 35 cycles of 95 °C for 30s, 48 °C for 45s, and 72 °C for 1 min, and final elongation at 72 °C for 10 min. Fragments were purified and sequenced bi-directionally at Macrogen Inc, Korea.

Table 2- Current study data profile that had been used for the analysis of genetic diversity and phylogeography of *P. argentipes* in Sri Lanka.

**Features of the analysis**	**Cytochrome Oxidase I**		**NADH dehydrogenase subunit 4**	**Concatenated data set (*COI* + *ND4*)**
	***COI* study alignment**	***COI* regional alignment**		
Accession Numbers	MZ882190 to MZ882255	MZ882190 to MZ882255, KC791432 to KC791437, HQ541166, HQ585366 to HQ585373, KT428789 to KT478792	MW002724 to MW002768, OK066156 to OK066198	Supplementary table
Number of sequences used for the analysis	148	167	133	121
Number of populations included in the analysis	5	7	5	5

Genetic diversity of *P. argentipes* in Sri Lanka

Sequences were assembled using DNA baser sequence assembler V 5.15 and searched against the GenBank database at the NCBI using BLAST to confirm the origin of the sandfly. The Open reading frames were observed using the ORF finder at NCBI [32]. Sequences were manually aligned with the ClustalW multiple alignment tool in MEGA 5.2 x, considering gaps as missing data. Haplotypes were generated using DnaSP v 5.10 [33]. All new haplotype sequences generated through this study were deposited in GenBank with accession numbers *COI*- MZ882190 to MZ882255 and *ND4*- MW002724 to MW002768, OK066156 to OK066198. The *COI* and *ND4* sequences were aligned at 1,427 bp – 2,960 bp and 8,162 bp – 9,494 bp positions, respectively, against the reference sequence (KR349298.1) of *P. papatasi* complete mitochondrial genome [34] as no any *P. argentipes* reference sequences available in databases. The *COI* and *ND4* alignment of the sequences were truncated to 455 bp and 598 bp respectively, by trimming the edges and primer binding sites (Table 3).

Intra-population genetic diversity based on *COI* and *ND4* sequences was analysed separately using the number of mutations, segregation sites, pairwise differences, haplotype number, haplotype diversity, nucleotide diversity, and nucleotide polymorphism index [35].

Phylogeographic analysis - Haplotype networks and phylogenetic relationships

The unique haplotypes for *ND4*, *COI* and concatenated (both *COI* and *ND4*) sequences were analysed for obtained sequences (*COI* - MZ882190 to MZ882255 and *ND4* - MW002724 to MW002768, OK066156 to OK066198) (Table 2).

The unique haplotypes for the Asia region (regional analysis) were analysed using previously published 19 *COI* sequences [36, 37] retrieved from GenBank deposits (KC791432 to KC791437, HQ541166, HQ585366 to HQ585373, and KT428789 to KT478792) and sequences from the current study (*COI* - MZ882190 to MZ882255) (Table 2). The relationship among haplotypes was evaluated by constructing independent median-joining haplotype

networks implemented in Network v 10.0 software [38] assuming epsilon equal to 0 and a transition/ transversion rate of 1:1.

Results

Sand fly collection, morphological identification, and DNA sequencing

Among the 159 collected *P. argentipes* individuals, 148 and 133 have given quality sequences for *COI* and *ND4* regions respectively. The concatenated sequence analysis used 121 sequences (Table 3).

Table 3: *COI* and *ND4* datasets and haplotype counts based on the number of segregation sites

Analysed gene fragments		Fragment size (bp)	Number of segregation sites	Number of haplotypes
**Cytochrome Oxidase I (*COI*)**	**A**	455	62	52
	**B**	343	37	36
**NADH dehydrogenase subunit 4 *(ND4)***		598	92	66
**Concatenated (*COI* and *ND4*) data set**		1053	140	84

(A) *COI* study alignment and (B) *COI* regional alignment

Genetic diversity of *P. argentipes* in Sri Lanka based on *COI* and *ND4* gene fragments

COI and *ND4* sequences were in sizes of 500 bp and 800 bp, respectively. *In silico* translations revealed no premature stop codons, ensuring that the sequences were free of nuclear mitochondrial DNA sequences (NUMTs).

The alignment of obtained *COI* sequences was composed of 62 polymorphic positions (13.62 %) (39 transitions and 23 transversions) with 35.20 % GC content, while *ND4* sequences showed 92 polymorphic sites (5.21%) (57 transitions and 35 transversions) with 27.4 % GC content (Table 4, S1 Table).

Table 4: Genetic diversity indices for *Phlebotomus argentipes* populations based on *COI* and *ND4* two gene fragments

**Parameters**	**Analysed Alignments**																
	**Cytochrome Oxidase I**												**NADH dehydrogenase subunit 4 *(ND4)***				
	**A**					**B**											
	**ANU**	**BAL**	**MIR**	**MED**	**HAM**	**ANU**	**BAL**	**MIR**	**MED**	**HAM**	**In**	**SL**	**ANU**	**BAL**	**MIR**	**MED**	**HAM**
**Number of sequences used**	25	31	28	24	40	25	31	28	24	40	13	6	27	29	21	25	31
**Number of sites**	455	455	455	455	455	343	343	343	343	343	343	343	598	598	598	598	598
**G+C content at coding positions**	0.352	0.352	0.352	0.352	0.352	0.347	0.347	0.347	0.346	0.346	0.344	0.346	0.274	0.274	0.274	0.274	0.274
**S**	19	19	13	15	27	7	14	8	9	10	3	4	20	23	22	57	38
**η**	19	19	13	16	29	7	14	8	10	11	3	4	20	24	23	62	42
**π**	0.00406	0.00348	0.00232	0.00354	0.00417	0.00163	0.00281	0.00167	0.00263	0.00147	0.00135	0.00622	0.00453	0.00464	0.00483	0.01055	0.00517
**h**	14	14	9	11	18	7	11	7	7	9	4	2	17	18	12	21	18
**Hd**	0.81	0.8	0.55	0.75	0.75	0.43	0.55	0.39	0.45	0.36	0.42	0.53	0.94	0.93	0.91	0.98	0.83
**Number of segregation sites analysed**	19	19	13	14	25	7	14	8	8	9	3	4	20	22	21	52	34
K_1	3.818					5.719							8.986				
K_2	4.174					5.225							4.633				
R	1.937					2.559							2.235				

(A) *COI* study alignment and (B) *COI* regional alignment, (In) GenBank retrieved, India *COI* sequence set, (SL) GenBank retrieved, Sri Lanka previous study *COI* sequence set, S- Number of variable sites, η - Total number of mutation, π- Nucleotide diversity, h- Number of haplotypes, Hd- Haplotype diversity, K_1 and K_2 are the transition/transversion bias rate ratios of purines and pyrimidines respectively, *R*- overall transition/transversion bias.

There were 52 haplotypes (Table 3) identified from *COI* alignment and 66 haplotypes identified for *ND4* with a range of 12- 21 haplotypes per site. According to the analysed data, there is a low level of genetic diversity for both mtDNA regions *COI* and *ND4*, with the range of π being 0.004- 0.002 and 0.01- 0.004, respectively (Table 4). The highest haplotype diversity (Hd) and nucleotide diversity (π) for both gene fragments were recorded from Medirigiriya (Hd- 0.977, π- 0.01055) and Hambantota (Hd- 0.749, π- 0.00417) respectively.

Genetic diversity of *P. argentipes* based on *COI* regional data set

GenBank retrieved *COI* sequences of *P. argentipes* (regional alignment) were compacted to 343 bp. The alignment consisted of 37 polymorphic sites (15 transitions and 22 transversions) with 4.66 % nucleotide polymorphism and 34.6 % GC content (Table 4, S1 Table). Thirty-six haplotypes were reported from 167 sequence alignments (Table 3).

Phylogeographic relationships of *P. argentipes* based on *COI* and *ND4* fragments.

COI

About 60.30 % of total mutation types recorded in *COI* sequences generated during the present study showed transitions, and the most frequent transition was Thymine - Cytosine (T-C 41.26%) (S1 Table). The frequent transversions comprised Adenine- Cytosine, and Guanine- Thymine. The Transition to transversion ratio was approximately 3:2. The most frequent haplotype, *HC1* was reported from all sampling localities, and was determined as the ancestral haplotype in Sri Lanka. Six haplotypes (*HC2, HC9, HC12, HC15, HC17* and *HC34*) have shared within 2-5 locations (Figure 3). Unique haplotypes were observed from all collection localities; the highest private haplotype count from Hambantota compared to the other four sampling localities (Anuradhapura (n=10), Balangoda (n=9), Mirigama (n=5) Medirigiriya (n=6) and Hambantota (n=13)) (Figure 3). All haplotypes have separated into nine divergent haplogroups (Figure 3, S2 Table) according to the median-joining

network analysis where topology does not coincide with the geographic distribution of the sampling localities.

Figure 3- Median-joining network for 148 *Phlebotomus argentipes COI* sequences in the current study.

Circle size and circle color indicate the haplotypes' frequency and geographical location, respectively. Haplotype labels are written next to the corresponding circles.

ND4

In *ND4* alignment, 56.07 % of point mutations were observed as transitions while Adenine - Guanine (A-G) was the most frequent. The highest abundant transversion was Adenine - Thymine (A-T 18.69%) (S1 Table). The transition to transversion ratio was approximately 9:7. The most frequent haplotype, HN1, was shared by all sampling localities and identified as the ancestral haplotype. Nine haplotypes (*HN2, HN6, HN8, HN10, HN12, HN16, HN19, HN20,* and *HN32*) were shared within 2-5 locations (Figure 4).

Private haplotypes were observed from all locations for the *ND4* region of *P. argentipes* (Anuradhapura (n=10), Balangoda (n=11), Mirigama (n=7), Medirigiriya (n=15) and Hambantota (n=12)) (Figure 4). All haplotypes have divided into three divergent haplogroups (S3 Table) according to the median-joining network analysis where the topology is absolved of the geographic distribution of the sampling localities.

According to the concatenated data set, four haplogroups (Table 5, S4 Table) were identified through median-joining network analysis.

Table 5- Identified genetic clusters and haplogroups of *Phlebotomus argentipes* in Sri Lanka based on *ND4* and *COI* fragments and concatenated sequence set

Data set	Number of Haplogroups	Haplogroup IDs
COI study data set	9	I, II, III, IV, V, VI, VII, VIII, IX
Regional *COI* study data set	5	X, XI, XII, XIII, XIV
ND4 study data set	3	XV, XVI, XVII
Concatenated (*COI* and *ND4*) data set	4	XVIII, XIX, XX, XXI

Figure 4- Median-joining network for 133 *Phlebotomus argentipes* study *ND4* sequences

Circle size and circle color indicate the haplotypes' frequency and geographical location, respectively. Haplotype labels are written next to the corresponding circles, red spots- median vectors.

Phylogeographic relationships of *P. argentipes COI* regional data set

The most frequent haplotype of *COI (HC1)* was not reported from GenBank-retrieved sequences from India (Figure 5A). However, *HC53*, *HC54*, *HC55* and *HC56* were found among sequences of Indian origin, and *HC56* showed an ancestral relationship with *HC1* (Figure 5B). Haplotypes of all analysed *COI* sequences were grouped into five divergent haplogroups in regional *COI* alignment (Table 5, S5 Table), which showed relationships between the network topology and sampling localities. All previously studied *COI* sequences of *P. argentipes* were from 2012 to 2014. Only four sequences (KC791437, KC791435, KC791433, and KC791432) have consisted of *HC1* haplotype in regional analysis. The present study sequences in the regional analysis are based on *P. argentipes* samples examined and collected from 2018 to 2020. The haplotype network for the regional *COI* data set emphasizes an isolation discrepancy between recent and previously studied haplotypes based on the period susceptible to evolutionary changes (Figure 5).

Figure 5- Median-joining network for 167 *Phlebotomus argentipes COI* sequences including GenBank retrieved sequences

Circle size and circle color indicate haplotypes' frequency and geographical location, respectively. Haplotype labels are written next to the corresponding circles (A) GenBank retrieved *COI* sequences/ regional alignment, (B) haplotypes derived from a previous study data set [36, 37].

Discussion

There is ample research worldwide on sandfly genetic variations and diversity (22), but the genetic diversity and population genetic structure for the *P. argentipes* are marginally explored. This study was focused on analyzing the genetic diversity of *P. argentipes* based on *COI* and *ND4* partial mitochondrial sequences.

Genetic diversity of *P. argentipes* in Sri Lanka

The nucleotide analysis of *P. argentipes* mtDNA revealed that *COI* and *ND4* possessed 64.80 % and 72.60 % A+T rich composition, respectively. It has been demonstrated that A+T rich composition in a species gives rise to the diminution of synonymous position and will devastate the amino acid content, affecting the substitution percentage of the amino acid [39].

When considering the haplotype analysis, both gene regions manifested high-overall haplotype diversity (Hd) (Hd> 0.5), but relatively low nucleotide diversity (π). However, according to the regional *COI* data set analysis, relatively higher Hd and π values were shown by Sri Lankan samples rather than by Indian counterparts. This has been attributed to the fragment length used for haplotype analysis developed due to many segregation sites [40]. Genetic variability of populations based on mtDNA markers are classified into four categories as (i) low Hd and low π (Hd < 0.5 and π < 0.005) (ii) high Hd and low π (Hd > 0.5 and π < 0.005) (iii) low Hd and high π (Hd < 0.5 and π > 0.005) (iv) high Hd and high π (Hd > 0.5 and π > 0.005) [41] and most of this study findings fall into the category (ii). One of the possibilities for such incidence could be population expansion after a period of a low effective population size followed by sudden population growth, which enhances the retention of new mutations as shown in [42, 43]. In other words, lower genetic diversity could be due to a population bottleneck followed by a reduction in effective population size in the past, which could be happened due to natural environmental events or human actions. Moreover, population bottlenecks decreased genetic variation by random genetic drift, which can reduce genetic variability and fitness (Birungi and Munsterman, 2002). As with other insect vectors [44], *P. argentipes* may experience periodic bottlenecks due to changes in host abundance and breeding sites. Only Medirigiriya and Hambantota sampling sites which have been categorized as higher disease epidemicity areas in [6] have been included in the fourth category of genetic variability (high Hd and high π) relevant to *ND4*. This indicates a high level of divergence between haplotypes. In this study, high haplotype diversity was found at both studied loci, with numerous unique haplotypes, suggesting either that they have already been established long ago [45] or more sampling is needed to detect the current variation.

The transition/ transversion (ts/tv) ratio and overall ts/tv bias for *ND4* gene fragment (K1 = 8.986 for purines, K2 = 4.633 for pyrimidines, and R= 2.235) are higher than *COI* (K1 = 3.818 for purines, K2 = 4.174 for pyrimidines, R= 1.937) (Table 3). This is attributed to post-mutation; the process occurs due to the high mutation rate of methylated cytosines to thymines in *ND4* than *COI* [46]. In addition to the high mutation rate and speed, *ND4* has many other advantages, including predefined marker systems, and PCR primers known to work for sandflies [14, 47, and 49]. Comparatively, most of the phylogeographic studies had been carried out based on the *ND4* than *COI* of mtDNA among these two markers due to the above facts [50].

COI and *ND4* partial sequence-based Haplotype distribution of *P. argentipes*

The *P. argentipes* population under study appeared to acquiesce to an extensive geographic transition by falling into nine haplogroups based on the *COI* region. The median-joining network analysis of the *COI* and *ND4* fragments identified the *HC1* and *HN1* as the ancestral haplotype, respectively. It presented and shared between five populations nucleated to the center of the radiating network. Other than the ancestral haplotype, the remaining haplotypes were differentiated from the ancestor by one to three mutational steps, fanned out radially. Most of the mutated haplotypes were received from the Hambantota site since the shared haplotypes were resident at the external nodes. The presence of many unique haplotypes may indicate that their populations are historically established. *P. argentipes* population in Sri Lanka has dispersed over a mosaic terrain and geographic pattern with expansion events, could be a reason for multiple haplogroups in different sites with relevance to *COI* and *ND4* sequences as described by Guernaoui *et al*, [15].

The patterns of haplogroups in all *COI* sequence analyses suggest that *P. argentipes* populations from widely separated and disjointed geographical areas are highly genetically differentiated, as shown by the differences in the frequencies of commonly occurring haplotypes. Two GenBank sequences, KC791436 and KC791434 from Punkuduthew and Delft islands of Northern Sri Lanka identified as geographically closer to the southern regions in India by analyzing KC791432 to KC791437,

HQ541166, and HQ585366 to HQ585373 sequences from South India, Kerala, and Tamil Nadu regions. The close association of the haplotypes mentioned above of Southern Indian and Northern Sri Lankan populations suggests persistent genetic contact, the probability of gene flow before marine introgression in the Miocene period, and slow evolution following the tectonic events. When considering the bionomics of the *P. argentipes* individuals, integration of the above factors, and the existence of Indian haplogroups in the northern regions of Sri Lanka due to ongoing gene flow/ migrations is a fact that couldn't explain or impracticable to explain by considering the bionomics of the sandfly. But the ancestral relationships among Indian and Sri Lankan *COI* haplogroups may have been established millions of years ago, based on only the historical geography of the ancient Indian Peninsula with Sri Lanka.

Conclusions

P. argentipes population in Sri Lanka is extremely homogenous, and the ancestor of the *P. argentipes* in Sri Lanka had evolved from India and was confirmed by the haplotype networks of mitochondrial DNA. *P. argentipes* is an evidently ancient, extremely adaptable species with a complex biogeographical history and possesses a suite of mitochondrial genes that appear to contain a record of the species' migratory history from India to Sri Lanka. The genetic homogeneity and ancestral history of *P. argentipes* in Sri Lanka were confirmed first by this study. This work presents new insights towards understanding the genetic diversity of *P. argentipes* in CL endemic areas in Sri Lanka. Further analyses will be needed to elucidate how different vector populations shape the genetic variability and population structure of *P. argentipes.* Furthermore, the genetic structure of both vector and parasite populations should be studied to get a deeper understanding of the Eco epidemiology of Sri Lankan cutaneous leishmaniasis.

Acknowledgments

The Epidemiology Unit, Ministry of Health, Sri Lanka, Anti- Malaria Campaign, and the regional public health officers of the sampling areas are acknowledged for providing leishmaniasis disease

prevalence data and providing assistance to the field works. Mr. Sanjaya Ranawaka, Survey department of Sri Lanka, is acknowledged for giving his support for Cartography works. The collaboration, support, and expertise provided by laboratory colleagues at the Molecular Genetic Laboratory, Centre for Biotechnology, are gratefully acknowledged for their contributions throughout this research.

References

1. Hartl DL, Clark AG, Clark AG. Principles of population genetics. Sunderland: Sinauer associates; 1997 Jan.
2. Frankham R. Genetics and conservation biology. Comptes Rendus Biologies. 2003 Aug 1;326:22-9.
3. Powell JR. Genetic variation in insect vectors: death of typology? Insects. 2018 Oct 11;9(4):139.
4. WHO- World Health Organization. Fact Sheets- Leishmaniasis [Internet]. 2023 [cited 26 Jun 2023]. Available: https://www.who.int/en/news-room/fact-sheets/detail/leishmaniasis
5. Centers for Disease Control and Prevention; Leishmaniasis - Biology [Internet]. 2023 [cited 2023 Apr 28]. Available from: https://www.cdc.gov/parasites/leishmaniasis/biology.html
6. Karunaweera ND, Ginige S, Senanayake S, Silva H, Manamperi N, Samaranayake N, Siriwardana Y, Gamage D, Senerath U, Zhou G. Spatial epidemiologic trends and hotspots of leishmaniasis, Sri Lanka, 2001–2018. Emerging infectious diseases. 2020 Jan;26(1):1.
7. Epidemiology Unit, Ministry of Health, Sri Lanka. Weekly Epidemiological Report [Internet]. 2023 [Cited June 26, 2023]. Available from: https://www.epid.gov.lk/weekly-epidemiological-report/weekly-epidemiological-report
8. Quintana MG, Fernández MS, Salomón OD. Distribution and abundance of phlebotominae, vectors of leishmaniasis, in Argentina: spatial and temporal analysis at different scales. Journal of Tropical Medicine. 2012 Jan 19;2012.

9. Neal AT, Ross MS, Schall JJ, Vardo-Zalik AM. Genetic differentiation over a small spatial scale of the sand fly *Lutzomyia vexator* (Diptera: Psychodidae). Parasites & vectors. 2016 Dec;9(1):1-9.
10. Lanzaro GC, Warburg A. Genetic variability in phlebotomine sandflies: possible implications for leishmaniasis epidemiology. Parasitology today. 1995 Apr 1;11(4):151-4.
11. Pech-May A, Ramsey JM, Gonzalez Ittig RE, Giuliani M, Berrozpe P, Quintana MG, Salomon OD. Genetic diversity, phylogeography and molecular clock of the *Lutzomyia longipalpis* complex (Diptera: Psychodidae). PLoS neglected tropical diseases. 2018 Jul 5;12(7):e0006614.
12. Flanley CM, Ramalho-Ortigao M, Coutinho-Abreu IV, Mukbel R, Hanafi HA, El-Hossary SS, Fawaz EE, Hoel DF, Bray AW, Stayback G, Shoue DA. Population genetics analysis of *Phlebotomus papatasi* sand flies from Egypt and Jordan based on mitochondrial cytochrome b haplotypes. Parasites & vectors. 2018 Dec;11(1):1-1.
13. Hamarsheh O, Presber W, Abdeen Z, Sawalha S, Al-Lahem A, Schönian G. Genetic structure of Mediterranean populations of the sandfly *Phlebotomus papatasi* by mitochondrial cytochrome b haplotype analysis. Medical and veterinary entomology. 2007 Sep;21(3):270-7.
14. Soto SI, Lehmann T, Rowton ED, Porter CH. Speciation and population structure in the morphospecies *Lutzomyia longipalpis* (Lutz & Neiva) as derived from the mitochondrial *ND4* gene. Molecular Phylogenetics and Evolution. 2001 Jan 1;18(1):84-93.
15. Guernaoui S, Hamarsheh O, Garcia D, Fontenille D, Sereno D. Population genetics of *Phlebotomus papatasi* from endemic and nonendemic areas for zoonotic cutaneous leishmaniasis in Morocco, as revealed by cytochrome oxidase gene subunit I sequencing. Microorganisms. 2020 Jul 6;8(7):1010.
16. Pesson B, Ready JS, Benabdennbi I, Martín-Sánchez J, Esseghir S, Cadi-Soussi M, Morillas-Marquez F, Ready PD. Sandflies of the *Phlebotomus perniciosus* complex: mitochondrial introgression and a new sibling species of *P. longicuspis* in the Moroccan Rif. Medical and Veterinary Entomology. 2004 Mar;18(1):25-37.
17. Hamarsheh O, Presber W, Yaghoobi-Ershadi MR, Amro A, Al-Jawabreh A, Sawalha S, Al-Lahem A, Das ML, Guernaoui S, Seridi N, Dhiman RC. Population structure and geographical subdivision of the

Leishmania major vector *Phlebotomus papatasi* as revealed by microsatellite variation. Medical and veterinary entomology. 2009 Mar;23(1):69-77.

18. Avise JC. Mitochondrial DNA polymorphism and a connection between genetics and demography of relevance to conservation. Conservation Biology. 1995 Jun 1;9(3):686-90.
19. Rokas A, Williams BL, King N, Carroll SB. Genome-scale approaches to resolving incongruence in molecular phylogenies. Nature. 2003 Oct 23;425(6960):798-804.
20. Esseghir S, Ready PD, Killick-Kendrick R, Ben-Ismail R. Mitochondrial haplotypes and phylogeography of *Phlebotomus* vectors of *Leishmania major*. Insect molecular biology. 1997 Aug;6(3):211-25.
21. Ishikawa EA, Ready PD, De Souza AA, Day JC, Rangel EF, Davies CR, Shaw JJ. A mitochondrial DNA phylogeny indicates close relationships between populations of *Lutzomyia whitmani* (Diptera: Psychodidae, Phlebotominae) from the rain-forest regions of Amazonia and northeast Brazil. Memórias do Instituto Oswaldo Cruz. 1999;94:339-45.
22. Hodgkinson VH, Birungi J, Quintana M, Deitze R, Munstermann LE. Mitochondrial cytochrome b variation in populations of the visceral leishmaniasis vector *Lutzomyia longipalpis* across eastern Brazil. American Journal of Tropical Medicine and Hygiene. 2003 Oct 1;69(4):386.
23. Belen A, Kucukyildirim S, Alten B. Genetic structures of sand fly (Diptera: Psychodidae) populations in a leishmaniasis endemic region of Turkey. Journal of Vector Ecology. 2011 Mar;36:S32-48.
24. Florin DA, Davies SJ, Olsen C, Lawyer P, Lipnick R, Schultz G, Rowton E, Wilkerson R, Keep L. Morphometric and molecular analyses of the sand fly species *Lutzomyia shannoni* (Diptera: Psychodidae: Phlebotominae) collected from seven different geographical areas in the southeastern United States. Journal of medical entomology. 2011 Mar 1;48(2):154-66.
25. Cohnstaedt LW, Caceres AG, Beati L, Munstermann LE. The population structure of Lutzomyia verrucarum (Diptera: Psycodidae), a *Bartonella bacilliformis* and *Leishmania peruviana* vector in Peru. Journal of medical entomology. 2012 Jan 1;49(1):77-84.

26. Dinesh DS, Das P, Picado A, Davies C, Speybroeck N, Ostyn B, Boelaert M, Coosemans M. Long-lasting insecticidal nets fail at household level to reduce abundance of sandfly vector *Phlebotomus argentipes* in treated houses in Bihar (India). Tropical Medicine & International Health. 2008 Jul;13(7):953-8.
27. Müller GC, Edita E, Beier JC. Simplified and improved monitoring traps for sampling sand flies. Journal of Vector Ecology. 2011 Dec;36(2):454-7.
28. Hoel DF, Kline DL, Hogsette JA, Bernier UR, El-Hossary SS, Hanafi HA, Watany N, Fawaz EY, Furman BD, Obenauer PJ, Szumlas DE. Efficacy of commercial mosquito traps in capturing phlebotomine sand flies (Diptera: Psychodidae) in Egypt. Journal of medical entomology. 2014 Dec 1;47(6):1179-84.
29. Lewis DJ. The phlebotomine sandflies (Diptera: Psychodidae) of the Oriental region. Bulletin of the British Museum (Natural History), B. Entomology. 1978;37(6):217-343.
30. Lane RP. Sandflies (Phlebotominae). Medical insects and arachnids. 1993:78-119.
31. Simon C, Frati F, Beckenbach A, Crespi B, Liu H, Flook P. Evolution, weighting, and phylogenetic utility of mitochondrial gene sequences and a compilation of conserved polymerase chain reaction primers. Annals of the entomological Society of America. 1994 Nov 1;87(6):651-701.
32. Wheeler DL, Barrett T, Benson DA, Bryant SH, Canese K, Chetvernin V, Church DM, DiCuccio M, Edgar R, Federhen S, Feolo M. Database resources of the national center for biotechnology information. Nucleic acids research. 2007; 36: 13-21.
33. Librado P, Rozas J. DnaSP v5: software for comprehensive analysis of DNA polymorphism data. Bioinformatics. 2009; 25(11):1451-2.
34. Ye F, Liu T, King SD, You P. Mitochondrial genomes of two phlebotomine sand flies, *Phlebotomus chinensis* and *Phlebotomus papatasi (Diptera: Nematocera),* the first representatives from the family Psychodidae. Parasites & vectors. 2015; 8(1): 1-3.

35. Rozas J, Ferrer-Mata A, Sánchez-DelBarrio JC, Guirao-Rico S, Librado P, Ramos-Onsins SE, Sánchez-Gracia A. DnaSP 6: DNA sequence polymorphism analysis of large data sets. Molecular biology and evolution. 2017; 34(12):3299-3302.

36. Gajapathy K, Peiris LB, Goodacre SL, Silva A, Jude PJ, Surendran SN. Molecular identification of potential leishmaniasis vector species within the *Phlebotomus (Euphlebotomus) argentipes* species complex in Sri Lanka. Parasites & vectors. 2013 ;6 (1):1-9.

37. Kumar NP, Srinivasan R, Jambulingam P. DNA barcoding for identification of sand flies (Diptera: Psychodidae) in India. Molecular Ecology Resources. 2012; 12(3):414-420.

38. Bandelt HJ, Forster P, Röhl A. Median-joining networks for inferring intraspecific phylogenies. Molecular biology and evolution. 1999; 16(1): 37-48.

39. Jukes TH, Bhushan V. Silent nucleotide substitutions and G+ C content of some mitochondrial and bacterial genes. Journal of Molecular Evolution. 1986 Dec;24:39-44.

40. Hamarsheh O. Distribution of Leishmania major zymodemes in relation to populations of *Phlebotomus papatasi* sand flies. Parasites & Vectors. 2011 Dec;4:1-6.

41. Grant WA, Bowen BW. Shallow population histories in deep evolutionary lineages of marine fishes: insights from sardines and anchovies and lessons for conservation. Journal of heredity. 1998 Sep 1;89(5):415-26.

42. Avise JC, Neigel JE, Arnold J. Demographic influences on mitochondrial DNA lineage survivorship in animal populations. Journal of Molecular Evolution. 1984 Jun;20:99-105.

43. Rogers AR, Harpending H. Population growth makes waves in the distribution of pairwise genetic differences. Molecular biology and evolution. 1992 May 1;9(3):552-69.

44. Birungi J, Munstermann LE. Genetic structure of Aedes albopictus (Diptera: Culicidae) populations based on mitochondrial ND5 sequences: evidence for an independent invasion into Brazil and United States. Annals of the Entomological Society of America. 2002 Jan 1;95(1):125-32.

45. Gómez-Romano F, Villanueva B, Rodriguez de Cara MA, Fernández J. Maintaining genetic diversity using molecular coancestry: the effect of marker density and effective population size. Genetics Selection Evolution. 2013 Dec;45(1):1-8.

46. Holliday R, Grigg GW. DNA methylation and mutation. Mutation Research/Fundamental and Molecular Mechanisms of Mutagenesis. 1993 Jan 1;285(1):61-7.

47. Depaquit J, Ferté H, Léger N, Killick-Kendrick R, Rioux JA, Killick-Kendrick M, Hanafi HA, Gobert S. Molecular systematics of the phlebotomine sandflies of the subgenus *Paraphlebotomus* (Diptera, Psychodidae, Phlebotomus) based on ITS2 rDNA sequences. Hypotheses of dispersion and speciation. Insect Molecular Biology. 2000 Jun;9(3):293-300.

48. Depaquit J, Ferté H, Léger N, Lefranc F, Alves-Pires C, Hanafi H, Maroli M, Morillas-Marquez F, Rioux JA, Svobodova M, Volf P. ITS 2 sequences heterogeneity in *Phlebotomus sergenti* and *Phlebotomus similis* (Diptera, Psychodidae): possible consequences in their ability to transmit Leishmania tropica. International Journal for Parasitology. 2002 Aug 1;32(9):1123-31.

49. Tsirigotakis N, Pavlou C, Christodoulou V, Dokianakis E, Kourouniotis C, Alten B, Antoniou M. Phlebotomine sand flies (Diptera: Psychodidae) in the Greek Aegean Islands: ecological approaches. Parasites & vectors. 2018 Dec;11:1-4.

Supporting Information

S1 Table. Parsimony Informative sites for *COI* and *ND4* data sets of *Phlebotomus argentipes* and the mutation positions of alignments had been marked by using the reference mtDNA genome of *Phlebotomus papatasi* KR349298.1.

S2 Table. Identified haplogroups in *COI* study data set

S3 Table. Identified haplogroups in *ND4* study data set

S4 Table. Identified haplogroups in concatenated study data set

S5 Table. Identified haplogroups in *COI* regional data set

Figure 1 Click here to access/download;Figure;Fig 1.jpg

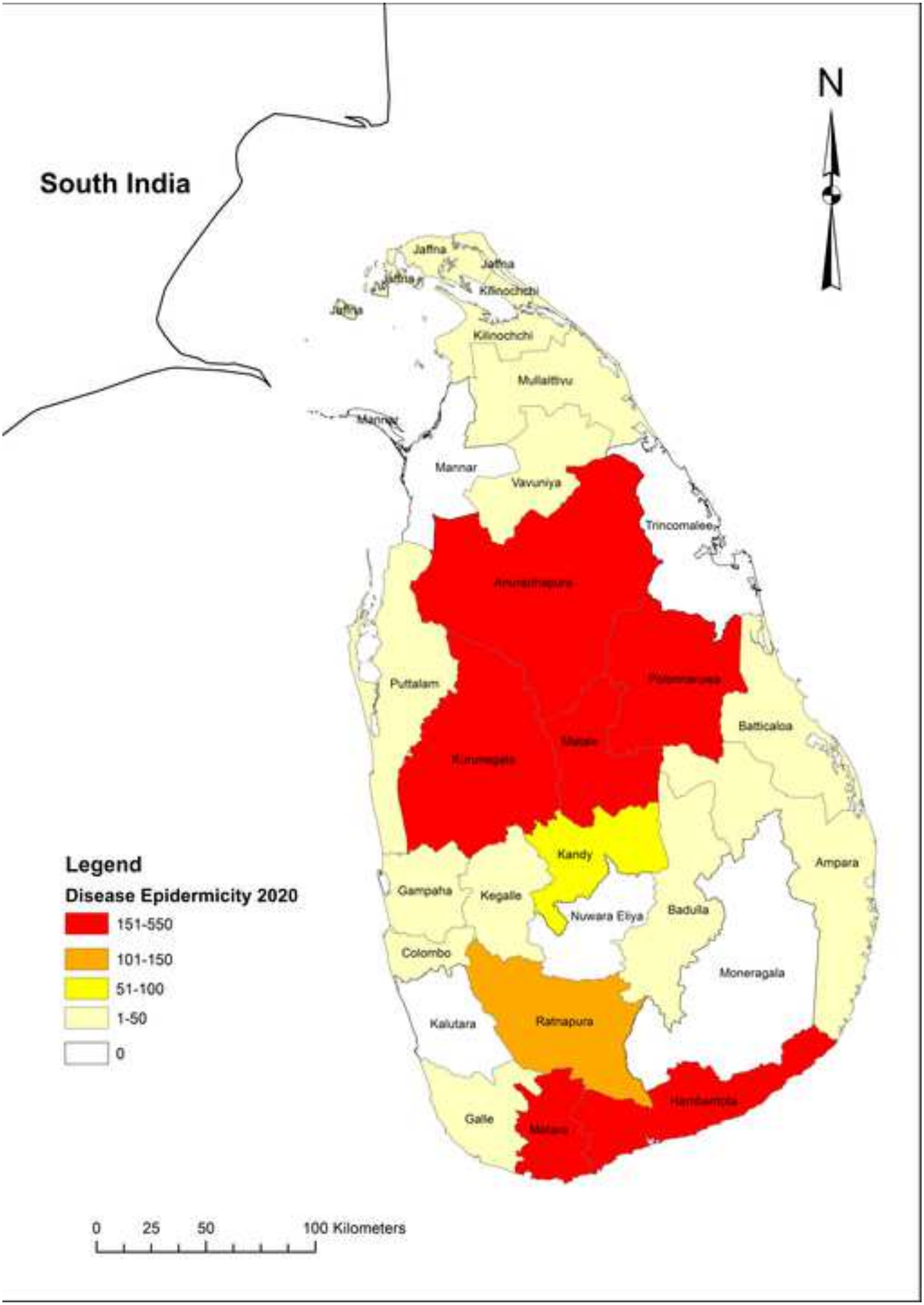

Figure 2 Click here to access/download;Figure;Fig 2.jpg

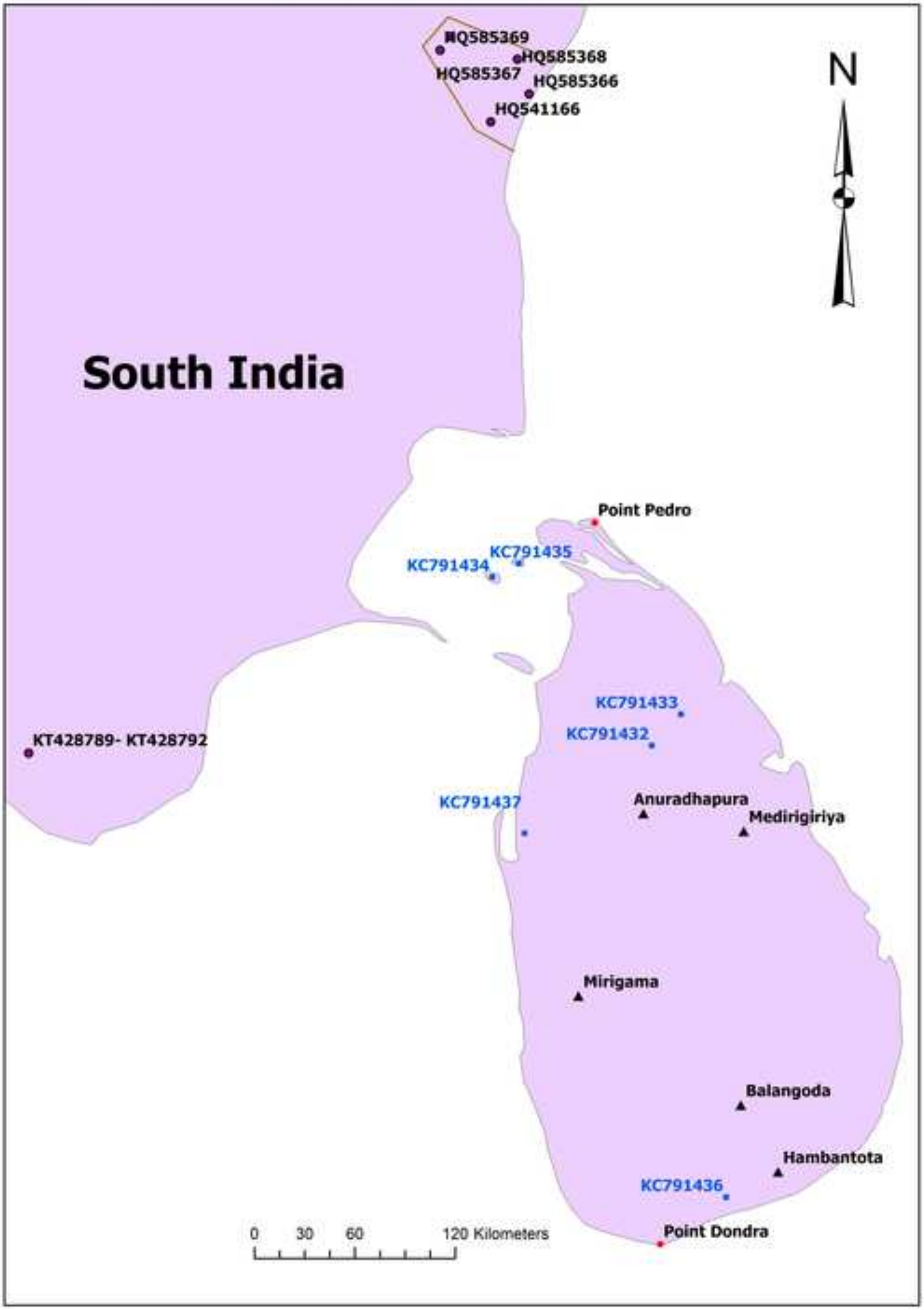

Figure 3

Click here to access/download;Figure;Fig 3.png

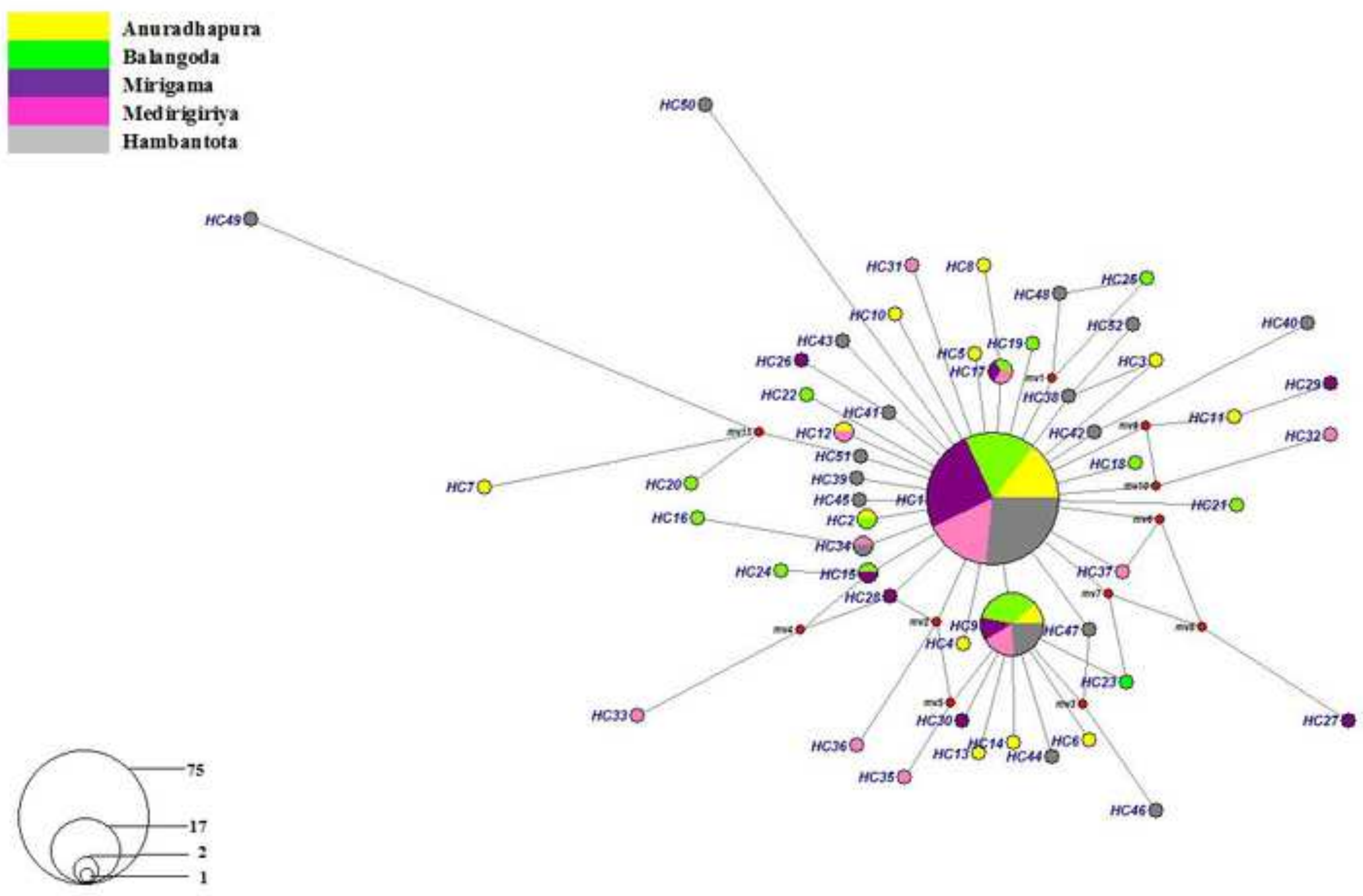

Figure 4

Click here to access/download;Figure;Fig 4.png

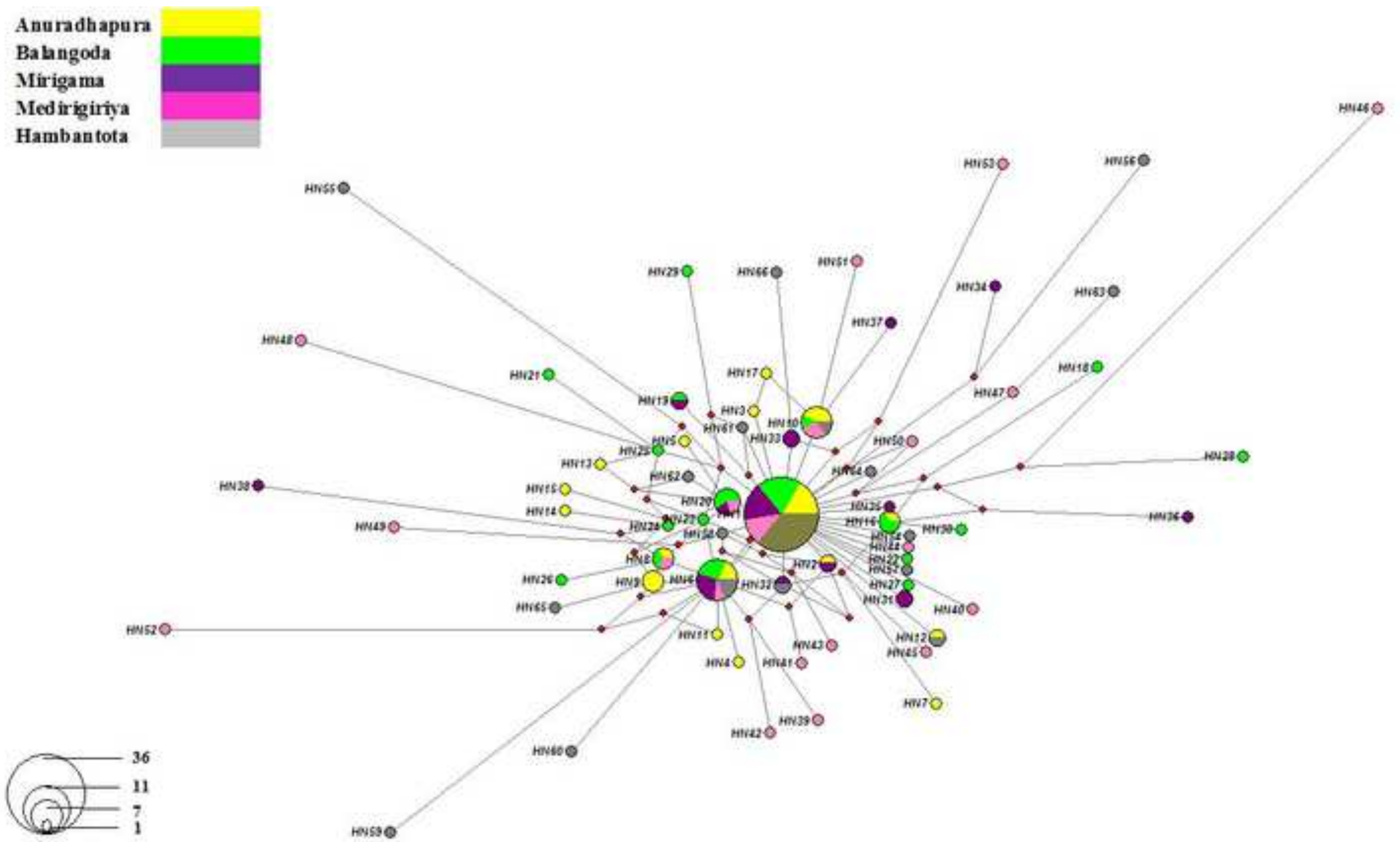

Figure 5

Click here to access/download;Figure;Fig 5.png

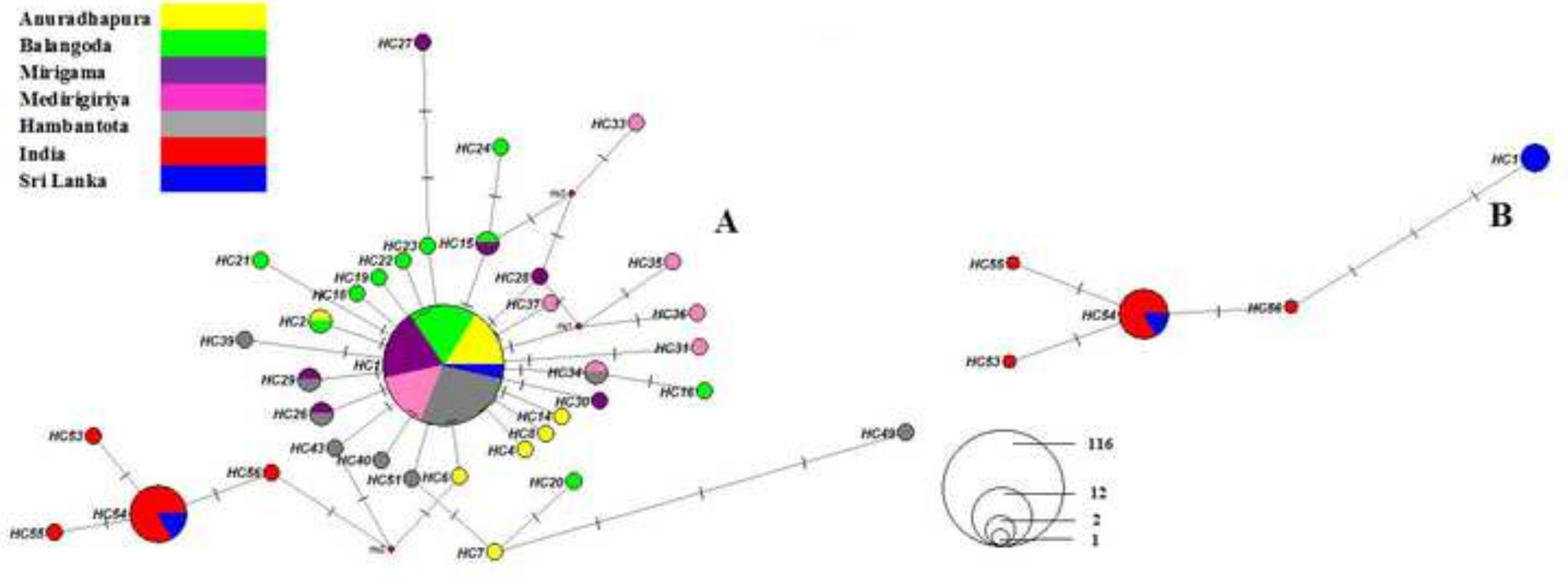

Click here to access/download
Supporting Information - Compressed/ZIP File Archive
Supporting Informations.zip
