# Supplementary material for: Genetic diversity and phylogeography of Phlebotomus argentipes (Diptera: Psychodidae, Phlebotominae), using COI and ND4 mitochondrial gene sequences
Source: PLoS One. 2023 Dec 29;18(12):e0296286. doi: 10.1371/journal.pone.0296286 (PMC10756540; doi:10.1371/journal.pone.0296286)
Supplement: Attachment — Submitted filename: Response to reviewers.docx [file pone.0296286.s011.docx]

**Response to reviewer comments**

**Reviewer 1-**

| Section | Comments | Response |
| --- | --- | --- |
|  | Reviewer #1: Dear Authors, Thank you for providing a detailed response letter for my suggestions and comments. I have no longer major comments about your manuscript. Best regards | We express our gratitude to the reviewer for their invaluable feedback, which has significantly contributed to the enhancement of this work. |

**Reviewer 2-**

| Section | Comments | Response |
| --- | --- | --- |
|  | Reviewer #2: The manuscript "Genetic diversity and phylogeography of Phlebotomus argentipes (Diptera: Psychodidae, Phlebotominae), using COI and ND4 mitochondrial gene sequences” provides relevant information on the genetic diversity. I thank the authors for considering my observations and including them in their work. I extend congratulations on your research achievements. Although the article has improved a lot and I believe it can be published, some spelling errors should be corrected first by the authors. I have attached my final comments. Best regards. | We express our gratitude to Reviewer 2 for providing valuable comments, feedback, and suggestions on our manuscript. These inputs have significantly contributed to the improvement of our work. We have implemented the suggested corrections as advised. |
|  | Line 52: delete extra parenthesis (ND4) | Extra parenthesis has been removed in the revised manuscript line no 52. |
|  | Line 107: P. argentipes | Mentioned correction has been done in the revised manuscript line no 107. |
|  | Line 127: delete extra parenthesis (ITS 2), and change parenthesis by brackets [cytochrome b (Cyt b), COI, and ND4], | Extra parenthesis has been removed in line no 127 and the mentioned brackets have been included in same line in the revised manuscript. |
|  | Line 160-161: A total of 159 specimens of P. argentipes were used for the present analysis. | Mentioned correction has been done in the revised manuscript line no 160. |
|  | Line 166-167: Identified sandfly specimens were preserved in 70% ethanol before extracting DNA. | Mentioned correction has been done in the revised manuscript line numbers 165- 166. |
|  | Line 176: please change individuals by “specimens” | The word “individuals” has been replaced with “specimens” in the whole document with relevance. |
|  | Line 180-182: each primer C1J (5'- GGA GGA TTT GGA AAT TGA TTA GTT C-3') and C1N (5'- CCC GGT AAA ATT AAA ATA TAA ACT TC-3'), | Mentioned correction has been done in the revised manuscript line numbers 179- 181. |
|  | Line 185-186: each primer ND4ar (5’- AA(A/G) GCT CAT GTT GAA GC-3’) and ND4c (5’-ATT TAA AGG (T/C)AA TCA ATG TAA-3’), For these two comments, I suggest you put the oligos names first and then the sequence (between parentheses), as well as separate them by triplets. | Mentioned correction has been done in the revised manuscript line numbers 184- 186. |
|  | Line 203-204: …9,494 bp regions respectively, against the reference sequence (KR349298.1) of P. papatasi complete mitochondrial genome [39] as no P. argentipes reference sequences available in databases. | The sentence has been corrected in revised manuscript line numbers 202- 204. |
|  | Line 214-217: The unique haplotypes for the Asian region (regional analysis) were analyzed using our COI sequences and 19 COI sequences previously deposited in the GenBank repository from India [41] and Sri Lanka (KC791432 to KC791437, HQ541166, HQ585366 to HQ585373, and KT428789 to KT478792) [10]. | The sentence has been corrected in revised manuscript line numbers 213- 216. |
|  | Line 225: please change individuals by “specimens” | Word has replaced with “specimens” in revised manuscript line no 223. |
|  | Line 272-273: Circle size indicates the haplotype frequency, and the circle color the geographical location. Haplotype labels are written next to the corresponding circles. | The sentence has been corrected in the revised manuscript lines 270- 271. |
|  | Line 295-296: Circle size and color indicate the frequency and geographical location of the haplotypes respectively. Haplotype labels are written next to the corresponding circles, and the red circles indicate median vectors. | The sentence has been corrected in the revised manuscript lines 292- 294. |
|  | Line 312-315: Circle size and color indicate haplotype frequency and geographical location respectively. Haplotype labels are written next to the corresponding circles: (A) GenBank retrieved COI sequences/ regional alignment, (B) haplotypes derived from a previous study data set. | The sentence has been corrected in the revised manuscript lines 310- 312. |
|  | Line 365-368: The study of genetic markers such as the ND4 gene has allowed for a finer resolution in the analysis of genetic variability within P. argentipes populations. Identifying numerous unique haplotypes, this research contributes to understanding the intricate genetic landscape of this sandfly species in CL endemic regions of Sri Lanka. | Mentioned correction has been done in the revised manuscript line numbers 362- 365. |
|  | Line 381: cryptic species | The sentence has been corrected in the revised manuscript lines 378. |

**Reviewer 3-**

| Section | Comments | Response |
| --- | --- | --- |
|  | Reviewer #3: Line 96 The Leishmania protozoa in their intracellular form are not considered flagellates, as the flagellum in this form is almost imperceptible; therefore, the phrase becomes confusing. I suggest replacing it with "caused by protozoan parasites of the genus Leishmania. | We thank reviewer 3 for his/her valuable comments, feedbacks and suggestions on our manuscript. Below changes were made accordingly in the manuscript line no 96. |

**Response to editor comments**

| **Comment/ Suggestions** | **Response** |
| --- | --- |
| **PLOS ONE Decision: Revision required [PONE-D-23-20755R1] - [EMID:ae3de9bee523e054]** | |
| Please review your reference list to ensure that it is complete and correct. If you have cited papers that have been retracted, please include the rationale for doing so in the manuscript text, or remove these references and replace them with relevant current references. Any changes to the reference list should be mentioned in the rebuttal letter that accompanies your revised manuscript. If you need to cite a retracted article, indicate the article’s retracted status in the References list and also include a citation and full reference for the retraction notice | We thank the editor for their prompt review and valuable feedback. We have thoroughly reviewed the reference list for the manuscript, and we can confirm that none of the cited articles have been retracted. We have carefully examined each reference, and all of them are accurate and up-to-date. |
